# EEG-fMRI Based Information Theoretic Characterization of the Human Perceptual Decision System

**DOI:** 10.1371/journal.pone.0033896

**Published:** 2012-04-02

**Authors:** Dirk Ostwald, Camillo Porcaro, Stephen D. Mayhew, Andrew P. Bagshaw

**Affiliations:** 1 Department of Neurology and Bernstein Center for Computational Neuroscience, Charité, Berlin, Germany; 2 Institute of Neuroscience, Newcastle University, Newcastle upon Tyne, United Kingdom; 3 School of Psychology and BUIC, University of Birmingham, United Kingdom; 4 LET's – ISTC – CNR, Ospedale Fatebenefratelli, Isola Tiberina, Rome, Italy; Max Planck Institute for Human Cognitive and Brain Sciences, Germany

## Abstract

The modern metaphor of the brain is that of a dynamic information processing device. In the current study we investigate how a core cognitive network of the human brain, the perceptual decision system, can be characterized regarding its spatiotemporal representation of task-relevant information. We capitalize on a recently developed information theoretic framework for the analysis of simultaneously acquired electroencephalography (EEG) and functional magnetic resonance imaging data (fMRI) (Ostwald et al. (2010), *NeuroImage* 49: 498–516). We show how this framework naturally extends from previous validations in the sensory to the cognitive domain and how it enables the economic description of neural spatiotemporal information encoding. Specifically, based on simultaneous EEG-fMRI data features from n = 13 observers performing a visual perceptual decision task, we demonstrate how the information theoretic framework is able to reproduce earlier findings on the neurobiological underpinnings of perceptual decisions from the response signal features' marginal distributions. Furthermore, using the joint EEG-fMRI feature distribution, we provide novel evidence for a highly distributed and dynamic encoding of task-relevant information in the human brain.

## Introduction

The modern metaphor for the human brain is that of a dynamic, information processing device [1]. By means of its neural activity, the brain is thought to represent information about the external state of the world, internal expectancies about incoming sensory information, as well as the formation and execution of decisional processes [2], [3]. A first step in understanding how neural activity represents this set of variables is to quantify the spatiotemporal dynamics of information representation in the brain. It is generally believed that knowledge about the information-carrying features of neuronal activity will lead to a better understanding of the dynamic principles that underlie brain function in health and disease.

A promising new methodology to accumulate this knowledge non-invasively from human observers carrying out cognitive tasks is the simultaneous recording of EEG and fMRI data (hereafter referred to as EEG-fMRI) [4]. The technical limitations of acquiring EEG-fMRI data have been largely overcome, owing to the development of improved EEG recording hardware and gradient- and ballistocardiogram-artefact removal techniques [5], [6]. However, a remaining obstacle preventing the full exploitation of the potential spatiotemporal resolution of EEG-fMRI in identifying information-carrying features of cortical activity is the uncertainty about how to integrate the two modalities. To this end, recent work has underlined the importance of single-trial fluctuations in EEG and fMRI data features in terms of their information content regarding stimulation and task performance, and the effect of ongoing brain activity on evoked responses [7]–[10].

We have previously proposed an information theoretic framework for the quantification of single-trial variability in EEG-fMRI integration [11], [12]. Information theory, and its core quantity of mutual information, allows inferences about which neuronal activity features probabilistically discriminate between experimental variables of interest. As the calculation of mutual information is explicitly dependent on the estimation of the joint stimulus EEG-fMRI signal probability function from single-trial responses, an information theoretic approach has the potential to take advantage of the full data variance, while relaxing the linearity and Gaussianity assumptions of standard methods for EEG-fMRI integration by prediction [13]. Importantly, for the case of non-simultaneous EEG and fMRI recordings, the joint stimulus-EEG-fMRI signal distribution can only be approximated as a factorized joint distribution which provides the crucial motivation for analyzing EEG-fMRI data using an information theoretic approach [14].

**Figure 1:**
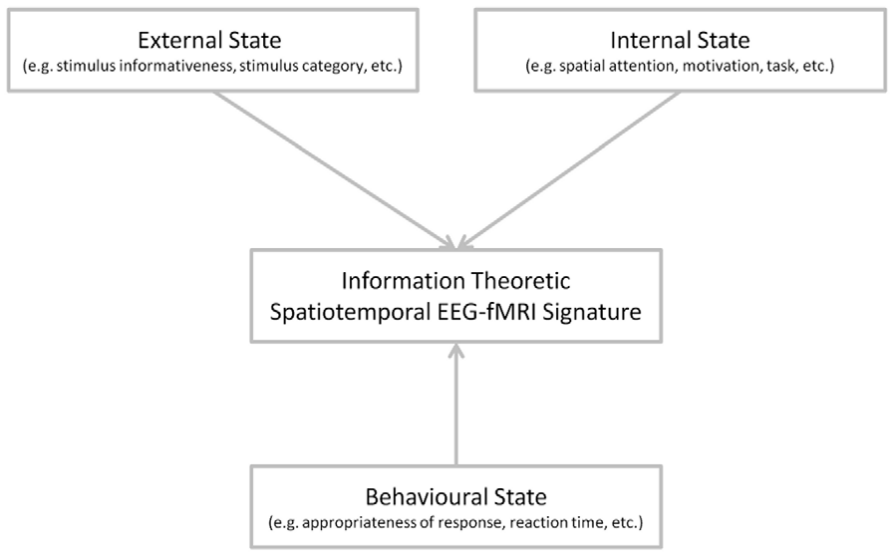
Conceptual Framework. A system view of the human brain implicates neuronal processes in the representation of information about external states (e.g., stimulus informativeness, stimulus category, etc.), internal states (e.g., spatial attention, task, motivation, etc.), as well as behavioural states (e.g., response appropriateness, reaction time, etc.). All state variables are assumed to contribute to an observed information theoretic spatiotemporal EEG-fMRI signature in a given neuroimaging experiment. For the current study, these variables have been operationalized as stimulus category and informativeness (external state), spatial attention/prioritization (internal state) and the observer's decision and response time on a given experimental trial (behavioural state).

Previously, this framework has been validated based on EEG-fMRI recordings of passive sensory stimulation, i.e. identifying informative neural activity features about an external variable. In the current study we investigated how the information theoretic framework for EEG-fMRI data analysis can be applied to questions of cognitive neuroscience, involving external as well as internal and behavioural experimental variables (Figure 1). Specifically, we show how the proposed framework naturally extends to the cognitive neuroscience setting and exemplify its application using a visual perceptual decision paradigm with spatial attention modulation.

Perceptual decisions are arguably one of the core cognitive functions [15] and can be defined as the selection of one among a set of possible interpretations of a sensory event [16]–[19]. Previous research indicates that perceptual decisions are based on the accumulation of sensory evidence over time [20]–[23]. Electrophysiological research in primates has led to the concept of neural integrators thought to implement such accumulation [24], while functional brain imaging studies have identified areas potentially involved in the processing of perceptual decisions [16], [22], [25]–[27]. However, these areas, here referred to as the human perceptual decision system, have as yet not been characterized in a principled manner with respect to the information they represent about external stimulus variables (e.g. stimulus quality), decision-modulating internal states (e.g. spatial attention), nor behavioral variables (e.g. reaction times). The information theoretic framework for the analysis of EEG-fMRI data is highly suited to achieve this characterization as it capitalizes on a) the appropriate temporal and spatial scales of cortical activity, namely events on the millimeter and millisecond scale, and b) the appropriate ecological scale, namely the neural activity on the single-trial, based on which the brain is forced make a decision.

Finally, despite its many advantages, EEG-fMRI recording has detrimental effects on EEG data quality. While recent approaches allow the MR scanner induced EEG artefacts largely to be corrected, EEG data quality remains lower than for EEG recordings outside the MR environment. Given the subtle nature of the expected EEG effects, in addition to combined EEG-fMRI data acquisition, EEG data were also acquired for the same paradigm outside the MR environment. This allowed to determine the effect of poorer EEG quality on the information theoretic quantity patterns calculated from the combined EEG-fMRI data set.

In sum, the current study brings together the advances in EEG-fMRI recordings during performance of an ecologically meaningful cognitive task with the perspective provided by an information theoretic framework. While this approach results in methodological considerations that are perhaps more detailed than in standard reports on the neural underpinnings of perceptual decision making, we feel that these are necessary to allow the reader to assess how we are able to derive statements on the neuroscientific aspects of this study. Specifically, we demonstrate how the information theoretic framework is able to reproduce findings on the neural correlates of perceptual decisions based on the response-signal marginal distributions (i.e. based on unimodal EEG and fMRI data, respectively). Critically, we furthermore show how the high-dimensional EEG-fMRI data set can be collapsed economically onto spatiotemporal information surfaces summarizing the neurobiological underpinnings of perceptual decision making and thereby providing novel evidence for dynamical and distributed information encoding in the human brain.

All custom written Matlab (The Mathworks, Natick, MA) code used in this study is available from http://www.buic.bham.ac.uk/downloads/EEG_FMRI_ITQ/EEG_FMRI_PD_Analysis.zip and the data are available from the corresponding author upon request.

## Materials and Methods

### Subjects

Seventeen subjects (8 female, mean age 25.9 years, range 20–33 years, 2 left-handed) were recruited from the University of Birmingham campus and paid for their participation. All observers had normal or corrected to normal vision, no history of neurological disorders and gave written informed consent. The study was approved by the Science, Technology, Engineering and Mathematics Ethical Review Committee of the University of Birmingham.

Complete data sets (2 experimental runs of EEG alone, 5 experimental runs of simultaneous EEG-fMRI (see below)) were acquired from 13 of the 17 subjects (for two of the four subjects who did not have complete data, incomplete EEG data were recorded outside the MR scanner; for one of the four incomplete psychophysical data were recorded inside the MR scanner; while for the remaining subject the EEG data recorded inside the MR scanner were strongly contaminated by movement artefacts of the reference electrode due to contact with the head-coil). All information theoretic analyses (outside and inside MR scanner EEG data, fMRI data, and EEG-fMRI data) are based on the 13 complete data sets. To identify regions of interest for the information theoretic analyses with maximum detection power, a total of 16 fMRI data sets were included in the GLM-group analysis. One subject's fMRI data were excluded from the GLM analysis because no psychophysical data could be recorded.

### Experimental design and paradigm

**Figure 2:**
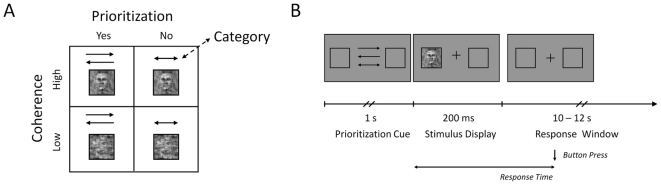
Experimental Design and Paradigm. *A. 2×2 factorial experimental design with factors informativeness (high, low) and spatial prioritization (yes, no)*. On each trial of the experiment, the observer was presented a face or car stimulus, which had been manipulated according to visual informativeness, and the observer was prompted to either spatially prioritize the stimulus display or not. The stimulus category (face or car), which the observer was asked to discriminate, was manipulated orthogonally to the other factors. *B. Single experimental trial outline*. Prior to the presentation of the stimulus, either a one-headed arrow indicated the hemifield of the subsequent stimulus presentation, or a two-headed arrow was uninformative in this respect. The cueing arrow was shown continuously for 1 s pre-stimulus, the stimulus itself for 200 ms. The observer was asked to respond as quickly and as accurately as possible with no restrictions on the response window. The inter-trial interval was 0–300 ms for the EEG only and 10–12 s for the combined EEG-fMRI recordings.

In a 2×2 factorial within-subject design, observers performed a perceptual decision task, similar to that described in [19], [22], [26] (Figure 2A). On each trial, a visual stimulus depicting either a face or a car was presented in one visual hemifield (left/right eccentricity of stimulus centre 11 degrees of visual angle, stimulus extension 9 degrees of visual angle) for 200 ms and the observer was asked to indicate via a button press whether a face or a car stimulus was presented. For the button presses, observers used their right index and middle finger for the two categories, and the mapping from stimulus category to response button was counterbalanced across observers. The informativeness of the visual stimulus was manipulated by altering the phase coherence of its spatial frequency spectrum resulting in low and high informative trials (see below). On half of the trials, a cueing arrow shown continuously for 1 s prior to the stimulus indicated in which hemifield the stimulus would be presented (Figure 2B). The observers were asked to allocate their spatial attention to the respective hemifield, while maintaining steady central fixation (spatial prioritization condition). On the other half of the trials, the two-headed cueing arrow was uninformative and the stimulus was presented randomly in either hemifield (no spatial prioritization condition). Face and car stimuli were equally distributed over the four experimental conditions. The order of stimulus presentation was randomized. Observers were asked to respond as quickly and accurately as possible with an emphasis on responding as quickly as possible and to maintain stable fixation of the central fixation cross throughout the experiment. Analyses of eye-movement data (Figure S1) obtained during the combined EEG-fMRI data acquisition in six subjects indicated that good fixation was achieved.

For the EEG only recordings outside of the scanner, data from 72 trials for each of the four conditions (half of them face stimuli) were recorded with an inter-trial interval randomized between 0–300 ms. The data acquisition was split into two experimental runs of approximately 10 minutes each. For the combined EEG-fMRI recordings data from 90 trials for each of the four conditions (half of them face stimuli) were recorded with an inter-trial interval discretely randomized between 10 s and 12 s (5 or 6 TRs). This long inter-trial interval was chosen to obtain reliable recordings of single-trial haemodynamic responses. The 90 trials were split into five experimental runs, each lasting approximately 14 minutes. Prior to the EEG recordings the observers also completed two practice runs to familiarise themselves with the task.

### Stimuli

The stimulus set consisted of 18 pictures of cars and 18 pictures of faces, similar to the stimulus set used in [19], [22], [26]. The car images were obtained from http://liinc.bme.columbia.edu/mainTemplate.htm?liinc_downloads.htm while the face images were obtained from the Max Planck face database [28]. The image categories were matched for the number of frontal, and left and right lateral views. All images were converted to bitmap format (.bmp) and the corresponding 256×256 matrices saved with 8 bit depth. The two stimulus sets were matched for their mean driving luminance and contrast as assessed by a one-way ANOVA with factor ‘image category’ and levels ‘face’ and ‘car’ (mean driving luminance: F_(1,34)_ = 0.08, p = 0.78, contrast: F_(1,34)_ = 0.22, p = 0.64). To manipulate the informativeness of the images, the spatial phase spectra were linearly weighted with a phase spectrum of a Gaussian noise image using the weighted mean phase technique as described in [29]. With the original phase of an image given by 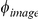, the final phase 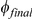 was computed as follows:
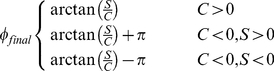
(1)where
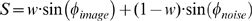
(2)

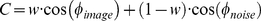
(3)and 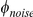 is the phase of uniform random noise and 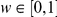 is the signal-to-noise weighting coefficient. Based on a psychophysical pilot study (Figure S2), stimuli with weighting coefficients 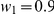 (high informativeness) and 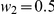 (low informativeness) were chosen for the experiment in order to elicit reliable differences in the response times for either stimulus class, while still allowing accurate performance of the task.

### Data acquisition

EEG data were recorded using a 64 channel MR compatible EEG system (BrainAmp MR Plus, Brain Products, Munich, Germany), which incorporates current limiting resistors of 5 kΩ at the amplifier input and in each electrode. The EEG cap consisted of 62 scalp electrodes distributed according to the 10–20 system [30] and two additional electrodes, one of which was attached approximately 2 cm below the left collarbone for recording the ECG, while the other was attached below the left eye (on the lower orbital portion of the orbicularis oculi muscle) for detection of eyeblink artefacts. Data were sampled at 5000 Hz. Impedance at all recording electrodes was less than 20 kΩ. For simultaneous EEG-fMRI recordings, the EEG data acquisition setup clock was synchronised with the MRI scanner clock using Brain Product's SyncBox, resulting in exactly 10,000 data points per EPI-TR interval (see details of the fMRI sequence below). The EEG set-up was identical for the recordings outside and inside the MR scanner. In the following, the EEG data set recorded outside the MR scanner will be referred to as EEG only, while the EEG data set recorded simultaneously with the fMRI data will be referred to as EEG-fMRI data.

The simultaneous EEG-fMRI experiment was conducted at the Birmingham University Imaging Centre using a 3T Philips Achieva MRI scanner. An initial T1-weighted anatomical scan (1 mm isotropic voxels) and T2*-weighted functional data were collected with an eight channel phased array SENSE head coil. EPI data (gradient echo-pulse sequence) were acquired from 32 slices (3×3×4 mm resolution, TR 2000 ms, TE 35 ms, SENSE factor 2, flip angle 80°). Slices were oriented parallel to the AC-PC axis of the observer's brain and positioned to cover the entire brain space.

Eye-movements were monitored for six observers while performing the task in the MR scanner using an ASL 6000 Eye-tracker (Applied Science Laboratories, Bedford, MA).

### Data pre-processing

EEG data acquired outside the MRI scanner were referenced to electrode FCz, partitioned into data acquisition sessions, band-pass filtered from 0.5 to 25 Hz and down-sampled to 500 Hz using Brain Vision Analyzer 2.0 (Brain Products, Munich, Germany). EEG data acquired inside the MR scanner were referenced to electrode FCz, partitioned into data acquisition sessions and the MR gradient and ballistocardiogram artefact removed using Brain Vision Analyzer 2.0, band-pass filtered from 0.5 to 25 Hz, and down-sampled to 500 Hz. To identify artefactual non-cerebral EEG components (i.e. eye-movements, muscular movement, environmental noise and, in the case of the EEG data acquired simultaneously with the EPI data, residual MR and BCG artefacts) a semiautomatic ICA-based procedure was employed [31], [32]. Upon rejection of the artefactual independent components and back-projection of the remaining independent components into channel space, all trials with maximum or minimum amplitudes outside a physiological range of −100 µV to 100 µV were discarded prior to further analyses.

SPM5 [33] was used for fMRI data pre-processing, and included anatomical realignment, slice scan time correction (reference slice 16), re-interpolation to 2×2×2 mm voxels, anatomical normalization to MNI space and spatial smoothing (5 mm FWHM Gaussian kernel).

### EEG data analysis

EEG data were analyzed in electrode space using custom-written Matlab code (The Mathworks, Natick, MA). Specifically, event-related potentials were computed using a 100 ms pre-stimulus baseline and 500 ms post-stimulus window. Because the stimuli were presented lateralized and occipito-parieto electrodes were considered of primary interest for the analysis, data for a given trial (left or right hemifield presentation) were allocated to the respective contra-lateral electrode set and collapsed over hemispheres for subsequent analyses. Grand averages of event-related potentials were computed across all trials of a given condition and subjects for pooled electrodes O1, O2, PO3, PO4, PO7 and PO8.

Upon the identification of time-windows of interest based on visual inspection of the grand average EEG data (see Results) and previous studies [19], [22], [27], [34]–[36], single-trial amplitude estimates were extracted from the EEG time-course for five discrete, non-overlapping time-windows of interest covering the entire −100 to 500 ms peri-stimulus period. For the EEG only data, these time-windows consisted of the intervals −100 to 58 ms, 60 to 120 ms, 122 to 154 ms, 156 to 370 ms, and 372 to 500 ms. As the equivalent neuronal and behavioural responses were slightly delayed for the combined EEG-fMRI data acquisition, the corresponding time-windows for the EEG-fMRI data were determined as −100 to 58 ms, 60 to 140 ms, 142 to 188 ms, 190 to 400 ms and 402 to 500 ms. For each time-window, except the third, the maximum amplitude on each single trial was extracted. For the third time-window, which encompassed a negative potential deflection, the minimum amplitude was extracted. These time-domain features were extracted from a set of eight parieto-occipital electrodes (O1/2, PO3/4, PO7/8, P1/2, P3/4, P5/6, P7/8 and TP7/8), whose selection was based on the topography of the grand average event-related potential (see Results). Upon feature extraction and information estimation, the information theoretic results for the EEG marginal features and EEG-fMRI joint features were averaged across these electrodes to yield the final information estimates.

### fMRI data analysis

To identify fMRI regions of interest (ROIs), the experimental data of each individual voxel was modelled using the standard univariate GLM approach in SPM5 [33]. A total of 16 experimental regressors were used, corresponding to the 8 stimulus conditions (2 coherence×2 prioritization×2 stimulus category levels) and 2 presentation locations (left and right visual hemifield). Voxel time-courses were modelled in an event-related fashion using regressors obtained by convolving each stimulus onset unit impulse with a canonical haemodynamic response function and its first temporal derivative. Additional nuisance covariates included the realignment parameters to account for residual motion artefacts and session specific means. A mixed-effects analysis was then implemented using a summary statistics approach to allow inferences at the population level [37], [38]: upon estimation of the model parameters for each subject, a subject-specific contrast image for each effect of interest was computed. Contrast vectors for the following effects of interest were used: all stimuli > fixation, left hemifield stimuli > right hemifield stimuli, right hemifield stimuli > left hemifield stimuli, high coherence stimuli > low coherence stimuli, low coherence stimuli > high coherence stimuli and face stimuli > car stimuli. The contrast images were then subjected to a one-sample t-test at the second level (group level).

For the information theoretic analysis filtered and whitened data were extracted from a sphere of 2 mm radius centred on the subject specific peak for the relevant contrast. The subject specific peak for each ROI was uniquely identified by visual inspection as the coordinates of the peak of the significantly activated cluster that was closest to the group mixed effects analysis coordinates. The average deviation across ROIs and observers from the group coordinates was 15 (±1 SEM) mm. Upon time-course extraction, single-trial event-related haemodynamic responses were computed as percent signal change with respect to a baseline comprising two pre-stimulus data points. The single-trial fMRI amplitude feature was then determined as the maximum over the 10 s post-stimulus period. The HRF amplitude was chosen as the only fMRI data feature as a) it was shown to be marginally more informative compared to the other fMRI data features in [11], b) the fMRI data features of [11] did not vary substantially, and c) to simplify the analysis and prohibit exponential growth in the number of EEG-fMRI data feature combinations.

It can be argued that an inter-stimulus interval of 10–12 s is too short to extract single-trial HRF maximum estimates without the use of an HRF deconvolution model. However, for the current stimulation protocol group average BOLD signal responses returned to baseline approximately 8–10 seconds post-stimulus and strong post-stimulus undershoots were not observed (data not shown, but see Figure S3 for a single subject example). Further, previous studies on the effect of the inter-stimulus interval indicate that inter-stimulus intervals can be reduced down to 6 s with very little change in HRF effect size [39], [40]. Given these considerations, extracting the single-trial HRF maximum amplitudes without deconvolution is appropriate in the context of the current study.

### Information theoretic EEG-fMRI feature integration

The calculation of information theoretic quantities from EEG-fMRI data features involves the estimation of probability mass functions, which in the current context was accomplished with a histogram approach as discussed below and in more detail in [41]. Here, only those aspects that are specific to the current experimental paradigm will be discussed.

To elucidate the spatiotemporal information representation signature for perceptual decisions, mutual information quantities were formulated relating to external, internal and behavioural state variables. Here, the external and internal variables are equivalent to experimental manipulations, i.e. stimulus variables. Specifically, the interest lies in a) the informativeness of the stimulus (spatial coherence), b) the stimulus category (face or car) and c) the observer's attentional state, parameterized by spatial prioritization. Let *S* denote the respective stimulus variable. The following quantities were computed with respect to the different response variable features
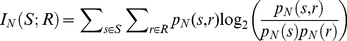
(4)where the variable *R* indicates either an EEG or fMRI data feature, and
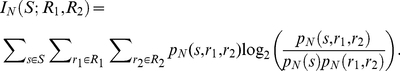
(5)where the variable *R_1_* indicates an EEG and the variable *R_2_* an fMRI data feature.

Intuitively, the quantity 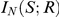 in equation (4) represents the relative distance of the observed stimulus-response joint distribution 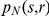 from its factorized counterpart 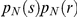 which embeds the assumption of stimulus-response variable independence for a univariate response feature. Similarly, 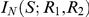 in equation (5) represents the same distance for a bivariate response feature, here comprising an EEG and an fMRI feature.

Analogously, with respect to the observer's behaviour, interest lies in 1) the subject's response time, and 2) the subject's categorical decision. Let *B* denote the respective behavioural variable, then
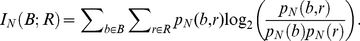
(6)where the variable *R* indicates either an EEG or fMRI data feature, and
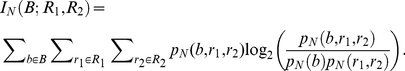
(7)where the variable *R_1_* indicates an EEG and the variable *R_2_* an fMRI data feature. The expressions with respect to the stimulus or the subject's behaviour are obviously analogous. However, it has to be noted that the marginal stimulus distribution 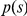 is usually determined by the experimenter and uniform, while the marginal behaviour distributions 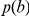 are experimentally observed, resulting in larger experimental uncertainty for the latter.

To estimate the information of a given EEG or fMRI feature or a feature combination about each of the variables of interest, the trials associated with this variable were sorted according to the respective variable categories and collapsed over all other stimulus categories. For example, to estimate 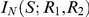 with respect to stimulus informativeness, trials were grouped into low stimulus spatial coherence 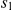 and high stimulus spatial coherence 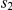, the joint observed probability distribution 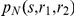 estimated and the informativeness of the signal features with respect to 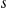 assessed. An analogous procedure was carried out for the information about the stimulus category (face vs. car) and about the observer's attentional state (spatial prioritization vs. no spatial prioritization). Figure S3 depicts an empirical example of the single-trial feature distributions for EEG and fMRI.

For the behavioural state variables, a similar procedure was carried out in a slightly modified manner: first, regarding the information about response times, all trials across all conditions were considered, and the joint probability distribution 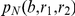 estimated, where *b* represents the continuous random variable response time. The small number of trials on which observers did not respond within 1 s of stimulus onset (average of 10.8% per subject) were excluded from the analysis to render the estimation of the joint probability distribution less prone to outliers (the histogram grid is adjusted to include the maximum and minimum values of each response variable, hence single outliers can have strong effects on the overall response space partitioning, which is to be avoided).

Finally, with respect to the observer's decision, only low coherence trials were considered in order to decouple the distribution of the observer's decision as much as possible from the physical stimulus category, i.e. 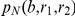 was estimated where *b* represents the distribution of the discrete random variable decision (face vs. car) on low spatial coherence trials. The current experimental paradigm was not optimized to study the informativeness of features with respect to the observer's perceptual state as the high accuracy of performance (see psychophysical results below) indicates that for most of the trials the physical stimulus category and the observer's perception matched. Future studies using near-threshold paradigms [42] might elucidate the informativeness of joint EEG-fMRI signal features about the observer's perceptual state in more detail. For the current study, it follows that the estimation of information about the observer's decision is more error prone compared to the other variables, as it proceeds based on half of the number of trials.

Based on the single-trial signal feature values extracted from the data, the respective probability distributions were estimated non-parametrically using a two-dimensional histogram approach with the number of bins set to 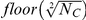, where *N_c_* denotes the number of trials per condition [43], [44]. Entropy and mutual information values were then computed using the respective equations and bias corrected for limited sample sizes using a combination of PT-, shuffling (1000 permutations)- and Gaussian null model (1000 simulations)-correction [41]. For expressions (6) and (7) the estimation of the marginal behavioural variable distributions is required. This entails a three-dimensional histogram analysis with the number of bins set to 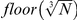 where 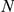 denotes the total number of trials evaluated. Given the non-Gaussianity of response time distributions [21] and the binary nature of the perceptual decision, the pure Gaussian null model bias correction employed for stimulus-response signal relationships was not appropriate. Hence, the respective null models for bias correction were based on sampling from three independent random variables (1000 simulations), two of which were Gaussian, representing the response signals, while the third, representing the behavioural variable, was either a Gamma distribution (response time null model) or a Bernoulli distribution (decisional variable null model).

The chosen numbers of response bins are relatively high and non-conservative, i.e. they maximize sensitivity to informative aspects in the data while decreasing specificity (i.e. increasing the risk of false-positives). Bias control procedures were employed to decrease the risk of false positives. However, the uncertainty about the absolute value of information in the current analysis is reflected in the fact that, in the following, only between-feature information comparisons are evaluated (i.e. the analysis focuses on relative information estimates) and no tests are performed for the difference of the information values from zero (i.e. the analysis does not focus on absolute information estimates).

Statistical comparisons of the estimated information quantities were carried out using one- or two-way repeated measures ANOVA models with Greenhouse-Geisser correction when appropriate, i.e. a significant result of Mauchy's test for sphericity followed by pairwise comparisons based on the estimated marginal means (least-significant differences) in SPSS (SPSS Inc, Chicago, IL).

## Results

In the following, traditional psychophysical, event-related potential and fMRI-GLM analyses are presented prior to the information theoretic analyses. These analyses serve the following purposes: 1) to make the reported IT results more comparable to similar studies of perceptual decisions, 2) to determine whether the experimental manipulations resulted in behavioural modulations, 3) to guide the identification of data features of interest, i.e. time-windows of interest for the EEG data and regions of interest for the fMRI data based on group results, and 4) to allow data quality assessment and inspection prior to single-trial feature distribution estimation.

Subsequently, the information represented in the data features of interest about the external, internal and behavioural variables of interest is presented successively for the EEG domain, the fMRI domain and finally the combined EEG-fMRI domain.

### Psychophysical results

**Figure 3:**
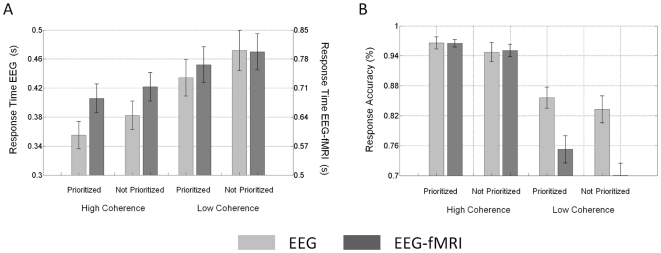
Psychophysical Results. *A. Response Times*. Bars depict the average (mean) median response times across observers, error bars ± standard error of the mean (SEM) *B. Response accuracy*. Bars depict the average (mean) median response times across observers, error bars ± standard error of the mean (SEM). The light grey bars (EEG) represent the EEG data set recorded outside the MR scanner, the dark grey bars (EEG-fMRI) represent the EEG data set acquired simultaneously with the fMRI data.

Figure 3 depicts the psychophysical results for EEG only and simultaneous EEG-fMRI experiments. In both cases, faster median response times were observed for high informative compared to low informative and spatially prioritized compared to not spatially prioritized stimuli (Figure 3A). Equivalently, response accuracies increased with stimulus informativeness and spatial prioritization (Figure 3B). The observed behavioural pattern was identical between the EEG only and simultaneous EEG-fMRI experiment. However, the MRI scanner environment lead to an overall increase in response times and decrease in performance accuracy (mean response time across conditions: 441 (±22 (Standard Error of the Mean (SEM))) ms EEG vs. 740 (±38 SEM) EEG-fMRI, accuracy across conditions: 90 (±2 SEM) % correct EEG vs. 84 (±2 SEM) % EEG-fMRI). Possible endogenous sources for this baseline shift to longer response times and lower accuracies include the noisy scanner environment, the uncomfortable scanning position, and fatigue, as the simultaneous EEG-fMRI data acquisition always followed the EEG data collection outside the MR environment. Possible exogenous sources include the lower quality of the visual projection as well as potential signal delays due to differences in the response button set-up and fibre optic conduction. Impairment in behavioural performance in psychophysical tasks for inside the MR scanner compared to outside the MR scanner have been reported previously (see [45] for a review).

To quantitatively assess the reliability of the experimental manipulation on the behavioural responses, a two-way repeated measures ANOVA with factors stimulus informativeness and spatial prioritization was carried out. For response times on correct response trials, this ANOVA revealed significant main effects of stimulus informativeness (EEG: F_(1,12)_ = 39.6, p<0.001, EEG-fMRI: F_(1,12)_ = 17.4, p = 0.001) and spatial prioritization (EEG: F_(1,12)_ = 12.1, p = 0.005, EEG-fMRI: F_(1,12)_ = 30.6, p<0.001). No significant interaction was observed (EEG: F_(1,12)_ = 1.1, p = 0.31, EEG-fMRI: F_(1,12)_ = 0.1, p = 0.81). For accuracy, significant main effects of stimulus informativeness (EEG: F_(1,12)_ = 54.9, p<0.001, EEG-fMRI: F_(1,12)_ = 146.7, p = 0.001) and spatial prioritization (EEG-fMRI: F_(1,12)_ = 7.8, p = 0.01) were observed, but not for spatial prioritization using the data recorded outside the MR scanner (F_(1,12)_ = 3.0, p = 0.10). There was no significant interaction (EEG: F_(1,12)_ = 0.1, p = 0.70, EEG-fMRI: F_(1,12)_ = 2,9, p = 0.11).

These results indicate that the experimental manipulation reliably evoked differential behavioural responses, while both experimental factors appear to act on independent cognitive substrates as no significant interaction was observed.

### Time course analysis of EEG data

**Figure 4:**
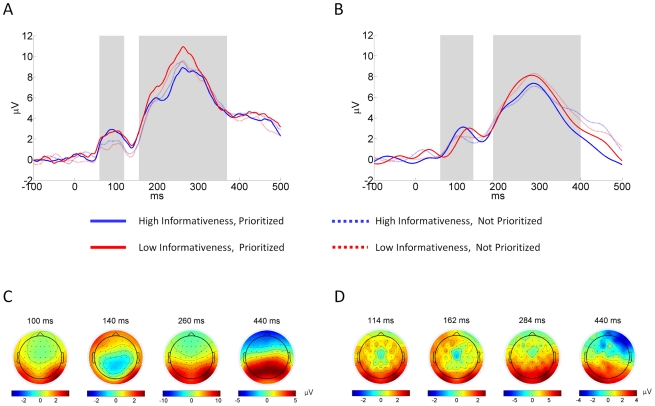
ERP analysis. *A and B. EEG grand average*. EEG grand average time-courses for contralateral trials pooled over electrodes electrodes O2, PO4, PO8 (left hemifield trials) and O1, PO3, PO7 (right hemifield trials) for the EEG only (*A*) and combined EEG-fMRI (*B*) data acquisition. The pattern of shaded and non-shaded areas reflects the five non-overlapping time-windows used for the subsequent information theoretic analyses. *C and D. EEG grand average topography*. Topography plots of the entire EEG electrode set show potentials for time-points 100, 140, 260 and 440 ms post-stimulus. The main positive deflections at all time-points were observed for a set of parieto-occipital electrodes (O2, O1, PO8, PO7, P8, P7, PO4, PO3).

To assess the data quality, to identify time-windows of interest, and to select electrode regions relevant for the current study, a traditional event-related potential (ERP) analysis was performed. Figure 4 depicts the grand average EEG time-courses for a set of parieto-occipital electrodes for both EEG only (Figure 4A) and combined EEG-fMRI data (Figure 4B). Given the hemifield presentation of the stimulus, these data were extracted from electrodes O2, PO4 and PO8 for left hemifield trials, from electrodes O1, PO3, and PO7 for right hemifield trials, and collapsed according to the experimental conditions. In line with similar previous studies [19], [22], no substantial potential deflections were observed after 500 ms post-stimulus, hence the focus of the analyses was on the −100 to 500 ms peri-stimulus time window.

With respect to the temporal expression of evoked EEG effects, for both data sets and all conditions, early (∼100 ms), and late (∼270 ms) positive potential deflections were identified (Figures 4A and 4B). These positive deflections, referred to as P100 and P300, respectively, were separated by an intermediate negative deflection (∼140 ms, N140), which was more prominent in the EEG only data. The most obvious condition-specific effects were an increase of the P100 amplitude with spatial prioritization and an increase of the P300 amplitude with a decrease of stimulus informativeness. These effects were clearly observed for both the EEG only and, with slightly diminished prominence, for the simultaneous EEG-fMRI data (Figures 4A and 4B). A clear stimulus condition specific effect on the N140 deflection was not observed.

Besides these stimulus evoked effects, we note that the EEG only data exhibited a higher degree of high frequency content than the simultaneous EEG-fMRI data. Identical filter settings were used during data pre-processing, so this temporal smoothing effect is likely due to the additional processing performed on the EEG-fMRI data (MR and BCG correction, ICA-based residual artefact removal) and the more efficient line-noise shielding of the MR scanner environment. Similar effects of artefact correction on the EEG power spectrum have previously been reported [46].

With respect to the spatial expression of evoked EEG effects, topography plots of the grand mean show that the strongest positive deflections for the identified time-points of interest were observed for posterior parieto-occipital electrodes (Figure 4, lower panels). These positive deflections were clearly visible for both the EEG only and simultaneous EEG-fMRI data. This motivated the joint selection of electrodes O1/2, PO7/8, P7/8, PO3/4, P5/6, TP7/8, P1/2 and P3/4 as spatial region of interest for the subsequent information theoretic analyses of both data sets. While the topography plots for the EEG only and EEG-fMRI data were in general similar, some differences remain. Specifically, we observed a smaller expression of the parietal dipole field at 140/162 ms and a weak leftward lateralization of the dipolar field at 440 ms for the EEG-fMRI compared to the EEG only data. These differences are likely due to residual artefacts in the EEG-fMRI data and possibly between-session effects. However, given the overall similar pattern of evoked effects for both data sets, the employed EEG-fMRI artefact correction has worked satisfactorily for the current purpose of evaluating relative information estimates for different data feature combinations.

In summary, the observed spatiotemporal pattern of peri-stimulus EEG responses motivated the information theoretic analysis of data extracted from parieto-occipital electrodes in five non-overlapping time-windows: 1) The pre- and early post-stimulus baseline, 2) the positive deflection around 100 ms (P100), 3) the negative deflection around 140 ms (N140), 4) the positive deflection around 270 ms (P300) and 5) the remaining time. These time-windows are indicated by the pattern of shaded and unshaded areas underlying the time-courses in Figure 4.

### General linear model analysis of fMRI data

To identify regions of interest for the subsequent information theoretic analyses a group level GLM analysis of the fMRI data set was performed. The aim of this analysis was to explicitly identify areas previously implicated in perceptual decision processes [17], [27], [47].

**Figure 5:**
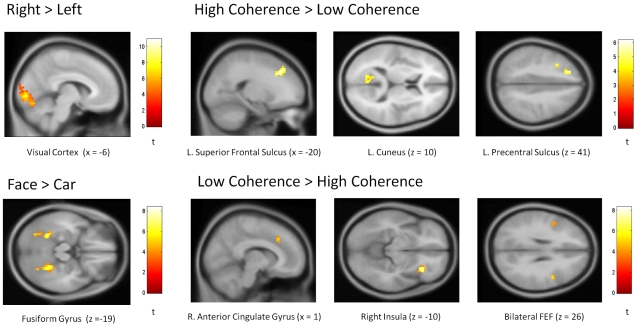
Group-level fMRI GLM results. The respective statistical parametric maps (thresholded at p<0.001 (uncorrected, voxel level), extent threshold 15–20 voxels) are overlaid on the group MNI template.

Figure 5 and Table 1 depict the results of the group-level GLM analysis of the fMRI data. Of all possible main effect contrasts, the main effect of prioritization was omitted as no significant activation was detected for this contrast at the group level. This was potentially due to the fact that the task demand (attention allocation) was high for both the prioritized and non-prioritized conditions. For the contrasts of left vs. right and right vs. left hemifield stimulus presentation, lateralized activity was detected in the occipital cortex, while higher level cortices did not display lateralized activity. This provides some validation for the use of single hemispheric signal features for occipital (striate cortex, extra-striate cortex, lateral occipital sulcus) regions of interest in the information theoretic analyses reported below.

**Table 1:** Group-level fMRI results.

Region	x	y	z	z-score peak	p-value
**Right > Left**					
L. Striate Cortex	−15	−81	−15	5.66	<0.001
L. Extra-Striate Cortex	−27	−72	−15	5.45	<0.001
L. Lateral occipital sulcus	−46	−80	10		
**Left > Right**					
R. Striate Cortex	12	−84	−3	5.32	<0.001
R. Extra-Striate Cortex	21	−75	−12	5.73	<0.001
R. Lateral Occipital Sulcus	51	−75	9	4.34	<0.001
**High Coherence > Low Coherence**					
L. Superior Frontal Sulcus	−21	27	39	4.22	0.001
L. Cuneus	−15	−69	12	4.29	<0.001
L. Pre-Central Sulcus	−33	12	45	3.79	0.083
**Low Coherence > High Coherence**					
R. Anterior Cingulate Gyrus	12	21	39	3.90	0.102
R. Insula	33	24	−9	5.01	<0.001
R. Frontal Eye Field	48	3	27	4.02	0.083
L. Frontal Eye Field	−45	6	24	3.40	0.156
R. Intra-Parietal Sulcus	30	−60	42	2.32	1.000
L. Intra-Parietal Sulcus	−24	−69	30	3.21	0.961
**Face > Car**					
L. Fusiform Gyrus	−27	−51	−24	5.04	<0.001
R. Fusiform Gyrus	27	−45	−21	4.74	<0.001

The MNI coordinates, z-scores of the cluster peak voxel and family-wise corrected p-values (cluster level) are displayed.

The set of regions identified as significantly activated (p<0.001 (cluster level, corrected)) for the high coherence vs. low coherence and low coherence vs. high coherence included superior frontal sulcus, pre-central sulcus, anterior cingulate gyrus, insula and frontal-eye fields, all of which have previously been implicated in the processing of visual perceptual decisions [16]. Additionally, given the known role of the intra-parietal sulcus in cognitive tasks [48], [49], the most active voxels in this region for the low vs. high coherence contrast were also identified and selected, although they were not significantly activated at the voxel level even at p<0.001 (cluster level, corrected). Finally, as observers performed a face vs. car categorization task, face responsive cortex of the fusiform gyrus (fusiform face area (FFA), [50]) was identified using the face vs. car stimulus contrast.

While not all of these regions reached family-wise error corrected statistical significance (Table 1) at the cluster-level, their previous implication in the perceptual decision process motivated their selection. As the motivation of the GLM analysis was to determine ROIs for the IT analysis, a relatively liberal threshold was used to avoid missing informative voxels.

### EEG-fMRI integrated information theoretic analysis

#### Temporal information representation

**Figure 6:**
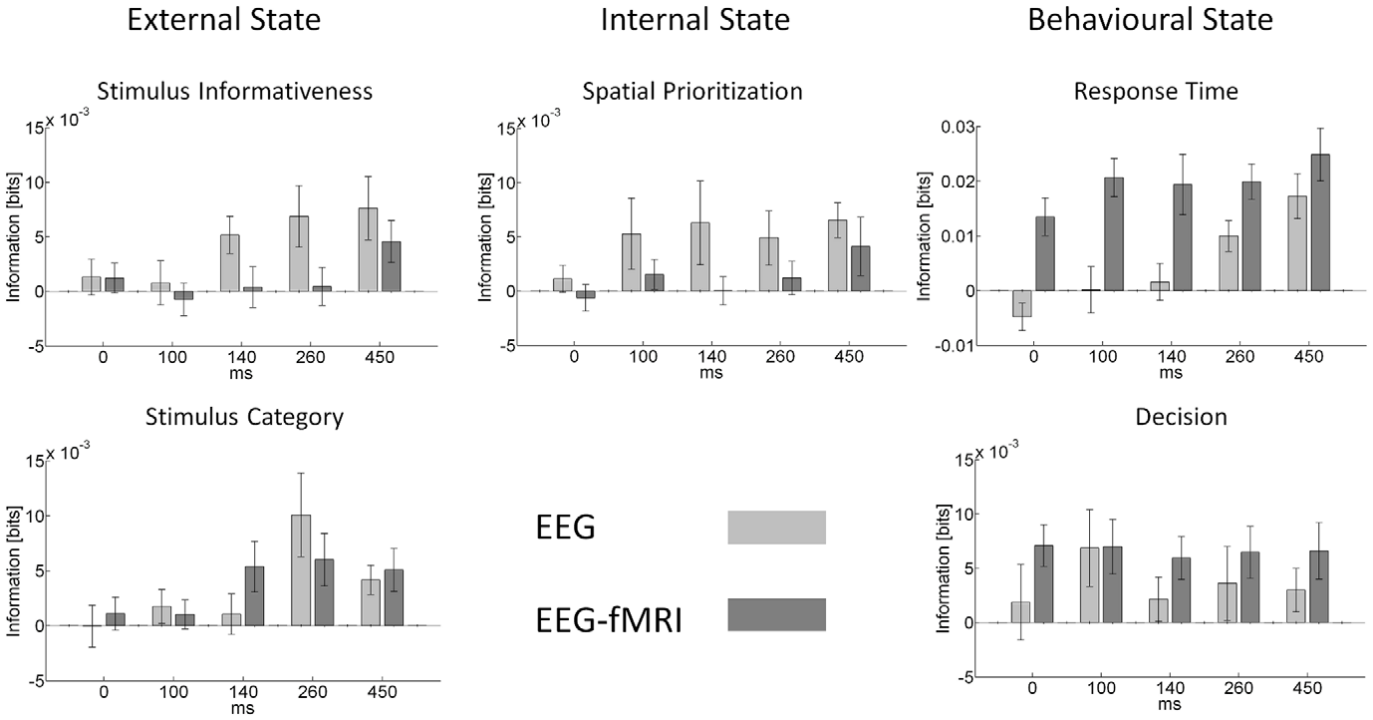
Temporal information representation. The data are ordered columnwise according to the variables of interest, external, internal and behavioural state. For each variable of interest, data from the five time-windows identified based on the grand average are displayed. The light bars represent information estimates 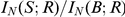 from the EEG data set acquired outside the MR environment, while the dark gray bars represent information estimates 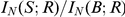 from the simultaneous EEG-fMRI data recordings. All bars reflect group averages (n = 13) and the error bars indicate the standard error of the mean.

Figure 6 displays the information about the external, internal and behavioural state variables for the EEG features of interest (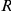). The columns of the figure represent the different stimulus and behavioural variables of interest (external, internal and behavioural state). For each stimulus/behavioural variable of interest (*S/B*), the average information estimate 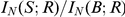 across subjects ± SEM is depicted for each of the five time windows of interest, in light grey for EEG only and in dark grey for the EEG-fMRI data sets. Overall, it can be observed that the information estimates for both EEG only and EEG-fMRI data sets, while being lower for the EEG data acquired simultaneously with fMRI data, show similar patterns. The lower information estimates for the EEG data acquired inside the MR scanner reflect its lower single-trial SNR.

For both data sets, the earliest time-window around stimulus onset yielded the lowest information estimates across features for most stimulus/behavioural variables, in agreement with the fact that information about the stimulus can only be extracted upon stimulus-presentation. With respect to the external state, the information about the stimulus informativeness increased from 140 ms onwards to reach its maximum in the final time-window. This effect was present in both data sets, although slightly diminished and delayed for the data acquired in the MR environment. For the EEG data acquired outside the MR scanner, a trend for a significant effect of the time-window on the information estimate was observed (F_(4,48)_ = 2.24, p = 0.07). The information estimate in the 450 ms time-window was marginally different from the information estimate in the 0 ms time-window (p = 0.06) and significantly different from the information estimate in the 100 ms time-window (p = 0.04). Similarly, for the EEG data acquired simultaneously with the fMRI data, a marginally significant main effect of time-window was observed (F_(4,48)_ = 2.42, p = 0.06), while the comparisons between the fifth and the first, second and third time windows approached statistical significance, or were statistical significant (p = 0.08, p = 0.03, and p = 0.02, respectively).

These results suggest that information about the stimulus informativeness, or equivalently, the difficulty of the decision, was represented rather late in the EEG response. This is in concordance with the observed grand average effect of stimulus informativeness depicted in Figure 4 and previous studies on the task-difficulty and reaction time sensitivity of the P300 deflection [27], [34]. It should be noted that for the current experimental design and analysis strategy, task difficulty, reaction time and P300 amplitude co-vary, and the activity dependent information theoretic analysis discussed above cannot dissociate these three concepts.

The other external stimulus attribute that was manipulated in the experimental paradigm was the stimulus category. Category-selective responses for faces compared to other stimuli have been described previously [19], [36]. In the current study, for both EEG data sets, the largest estimate for represented information about the stimulus-category was observed in the 260 ms time-window, i.e. in the interval of 150 to 370 ms post-stimulus onset. This is in concordance with the maximal discriminative time-windows for a similar stimulus set identified by [19]. This finding is substantiated by a significant effect of time-window on the information estimates for the EEG acquired outside the MR scanner (F_(4,48)_ = 6.01, p = 0.001) and statistically significant differences for the fourth time window in comparison to all others (p<0.05), except the fifth (p = 0.08). For the EEG data acquired inside the MR scanner, the main effect of time-window was not significant (F_(4,48)_ = 1.53, p = 0.20). However, the information estimate for the fourth time-window showed marginal statistically significant differences from that in the first and second windows (p = 0.05 and p = 0.06, respectively). The most information about the category of the stimulus was thus observed in a time-window 150–370 ms post-stimulus and declining thereafter.

In comparison to the information about the stimulus, the information estimates about the internal state, i.e. the spatial prioritization of the stimulus, were expressed earlier, with effects from 100 ms post-stimulus onwards. This is in line with the well-known attentional modulation effect on the P100 [35]. For the current data sets, the largest information about the attentional state of the observer was observed in the early time-windows of 100 and 140 ms and then, after a decrease in the fourth time-window, again in the last time-window. For the data recorded outside of the scanner, no statistically significant main effect of time-window was observed, consistent with the observation that the information estimates were similar for the time-windows from 100 ms on. The pairwise comparison between the fifth and the first time-window was marginally significant (p = 0.05), while the others were not. Again, for the EEG data acquired inside the MR scanner no significant main effect of time-window was observed, while the pairwise comparison between the fifth and the first time-window was the most reliable (p = 0.16).

Finally, with respect to behavioural state, for the response time the information estimates increased with time. This effect was significant for the EEG only dataset (F_(4,48)_ = 5.9, p = 0.001), but not for the EEG data acquired simultaneously with the fMRI data (F_(4,48)_ = 1.6, p = 0.18). This pattern is reminiscent of that observed for the information about stimulus informativeness, which is to be expected given the longer response times for low informative trials. Unfortunately, these two processes cannot be dissociated in the current paradigm. For the observer's decision, all EEG time-windows appear equally informative. Consistent with this observation one-way ANOVAs for both behavioural variables and EEG data sets indicated no statistically significant effects for both data sets (EEG only: F_(4,48)_ = 0.40, p = 0.80), EEG-fMRI: F_(4,48)_ = 0.04, p = 0.99). As the estimated information values for later time windows did not appear particularly different from those at the earliest time-point, it may be that the number of trials was too low or the electrode set chosen not appropriate, to detect a decisional effect on the basis of the marginal EEG data distributions. A possible reason for the observed larger information estimates for the EEG-fMRI data set is of methodological nature: while we aimed to reduce the number of outliers (see Methods), by design, the EEG-fMRI data set is more outlier prone with respect to behavioural variables. For our analysis this has the immediate effect that the partition of the data feature space is coarser than if all data feature combinations cluster in a smaller region of data feature space. However, coarser histogram sampling is known to entail larger estimation biases [51]. Future work on efficient outlier control, e.g. using the diffusion model framework [52], [53] may help to obtain better information estimates about the behavioural state.

In summary, the following picture of temporal representation of information for visual perceptual decisions emerges: in concordance with previous studies, information about the state of the stimulus is represented in the EEG response later than that about the subject's attentional state. With respect to behaviour, later time windows represent more information about the response time, in agreement with their involvement in the representation of uncertainty about the stimulus. The same patterns of results was observed for both data sets, although they were more reliable for the data recorded outside of the scanner. However, it is apparent that the observed single-trial information differences appear larger than those observed on the signals' grand averages. This motivates the future evaluation of novel methodologies for the improvement of EEG quality in combined EEG-fMRI recordings on the single-trial level [54].

#### Spatial information representation

**Figure 7:**
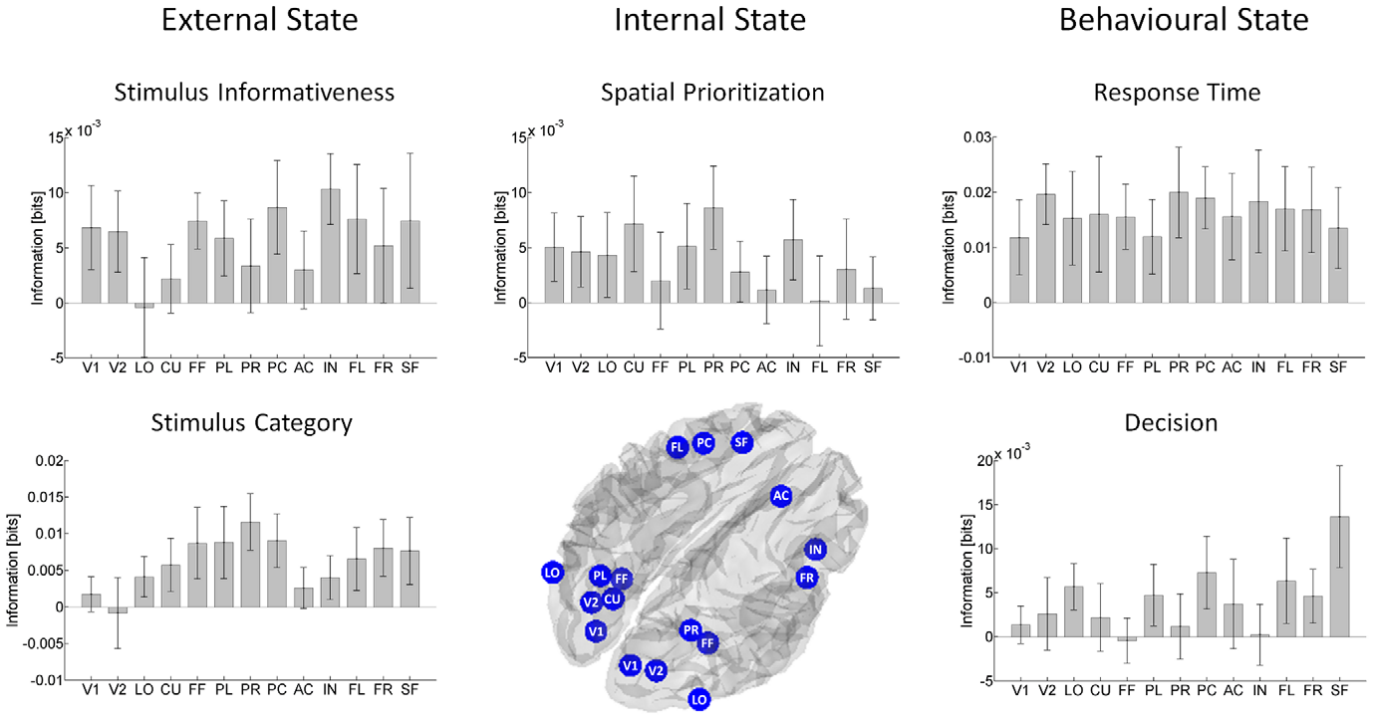
Spatial information representation. The data are ordered columnwise according to the variables of interest, external, internal and behavioural state. For each variable of interest, information estimates 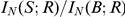 from all regions of interest identified based on the GLM group analysis are displayed. All bars reflect group averages (n = 13) and the error bars indicate the SEM. (V1: Striate Cortex, V2: Extrastriate Cortex, LO: Lateral Occipital Complex, CU: Cuneus, FF: Fusiform Gyrus, PL: Left Intra-Parietal Sulcus, PR: Right Intra-Parietal Sulcus, PC: Left Post-Central Gyrus, AC: Right Anterior Cingulate Cortex, IN: Right Insula, FL: Left Frontal Eye Field, FR: Right Frontal Eye Field, SF: Left Superior Frontal Gyrus).

**Figure 8:**
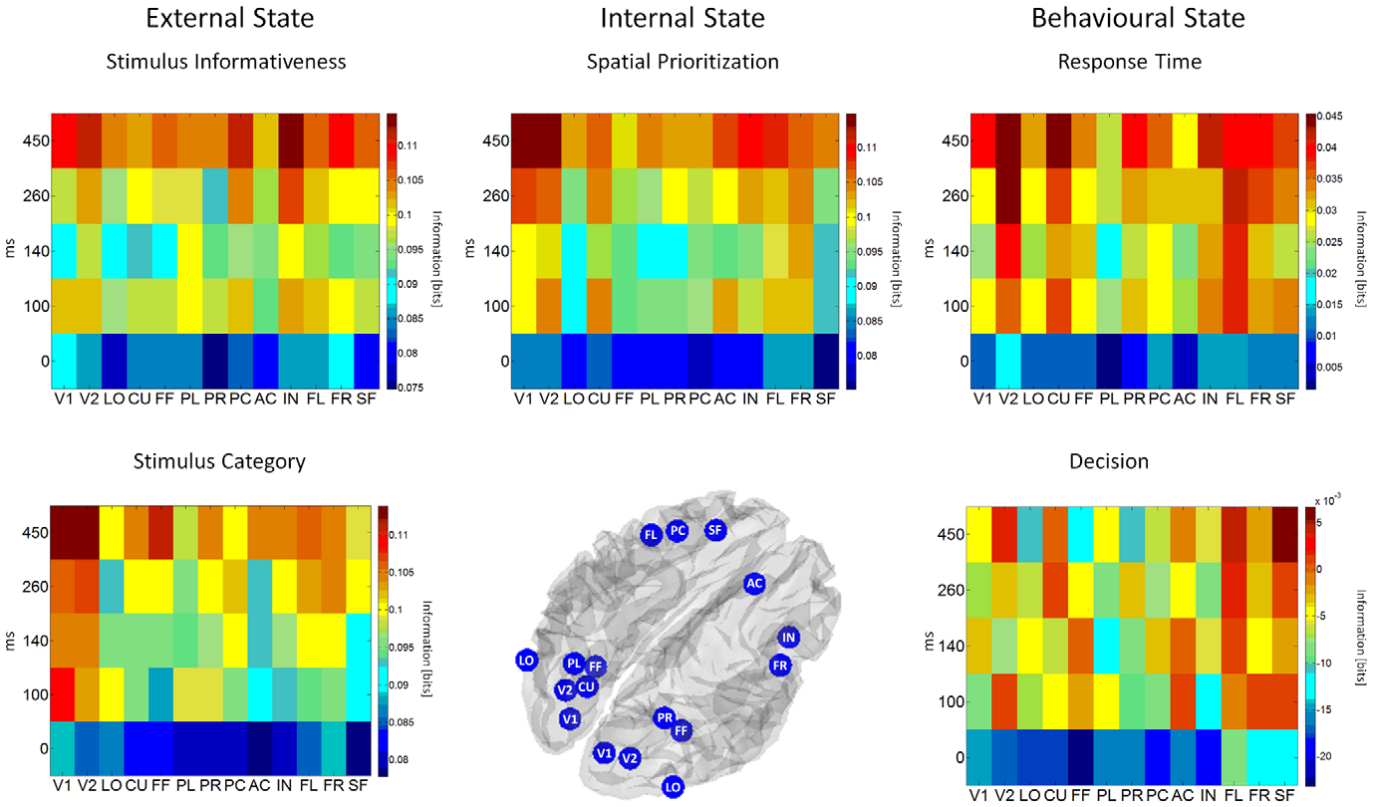
Spatiotemporal information representation. The data are ordered columnwise according to the variables of interest, external, internal and behavioural state. For each variable of interest, information estimates 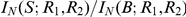 from all joint distributions of all features of interest combinations, i.e. EEG time-windows × fMRI regions of interest are displayed. The tiles of the surface reflect the group averages. (V1: Striate Cortex, V2: Extrastriate Cortex, LO: Lateral Occipital Complex, CU: Cuneus, FF: Fusiform Gyrus, PL: Left Intra-Parietal Sulcus, PR: Right Intra-Parietal Sulcus, PC: Left Post-Central Gyrus, AC: Right Anterior Cingulate Cortex, IN: Right Insula, FL: Left Frontal Eye Field, FR: Right Frontal Eye Field, SF: Left Superior Frontal Gyrus).

Figure 7 displays the information about the external, internal and behavioural state variables for each fMRI region of interest (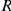). The columns of the figure represent the different stimulus and behavioural variables of interest (external, internal and behavioural state, *S*/*B*). For each stimulus/behavioural variable of interest, the average information estimate 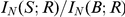 across subjects ± SEM is depicted for each of the regions of interest identified based on the group fMRI-GLM analysis. The regions of interest of each panel are ordered according to an approximate occipital - frontal (or “early sensory - higher cognition”) gradient from left to right, in line with Figure 8.

Overall, the information of relevance for the perceptual decision task employed in this study was spatially distributed across the cortex. Both anterior (higher) and posterior (lower) cortical areas were implicated in the representation of stimulus related information, while information about the internal state was slightly more strongly represented in posterior brain areas.

The areas primarily implicated in the representation of information about the stimulus informativeness or task difficulty comprise a network of frontal (insula, left frontal eye field, pre-central), parietal (left intraparietal sulcus) and occipital-temporal (extrastriate and fusiform) cortex. The most information about the stimulus informativeness was represented in insular cortex, with an estimate approaching a significant difference with respect to the cuneus and lateral-occipital cortex (p = 0.07, p = 0.04, respectively). Insular cortex has been implicated in perceptual decision making previously (e.g. [55]). Moreover, due to the involvement of insular cortex in a wide variety of cognitive processes (e.g. interoception, self-recognition, emotional awareness, time perception, attention, cognitive control and performance monitoring), it has been proposed that insular cortex plays a pivotal role in the neurobiological representation of awareness [56]. In the current study insular cortex appeared to be involved in the representation of task difficulty at the single-trial level, which could potentially be reconciled with this view, in the sense that insular cortex activity differentiates different states of the stimulus-dependent awareness induction.

Nevertheless, the distributed nature of the represented information is substantiated by the absence of an overall main effect of region of interest on the information about stimulus informativeness (F_(12, 144)_ = 0.78, p = 0.67). It should be noted that most areas were selected according to the contrast of high vs. low and low vs. high stimulus coherence, i.e. on the basis of being informative about stimulus coherence in the sense of a GLM contrast.

Categorical information about the stimulus was mainly represented in a network of frontal (superior frontal gyrus, frontal eye fields, pre-central) and parietal (intra-parietal sulcus (IPS)) regions, with some contribution from the fusiform gyrus. This finding is in concordance with the known roles of the IPS and superior frontal gyrus or dorso-lateral prefrontal cortex (DLPFC) in perceptual decisions [16], [17], [24]. While again the information appears to be distributed across regions of interest (F_(12, 144)_ = 1.01, p = 0.43), the pairwise comparison of the information estimate for the right IPS showed significantly higher information estimates in comparison to extrastriate visual (p = 0.03) and anterior cingulate cortex (p = 0.04).

A pair of occipito-parietal regions encompassing the cuneus and right parietal sulcus was most informative about the observer's state, i.e. the spatial prioritization of the stimulus. For the right intra-parietal sulcus, a post-hoc pairwise comparison of the information values across subjects reached marginal significance with respect to the right intra-parietal (p = 0.04), right frontal eye field (p = 0.04) and superior frontal gyrus (p = 0.06). This result is in line with previous studies using fMRI to study spatial attention [49], [57]–[60]. The involvement of a dorsal frontoparietal network of regions implicated in spatial attention in these studies was substantiated by the absence of a significant main effect of region of interest on information estimate (F_(5.4, 64.7)_ = 0.74, p = 0.60).

For the behaviour related information, no significant main effects of region of interest, or pairwise comparisons between regions were observed for response time (F_(5.0, 60.5)_ = 0.24, p = 0.94). The widespread network of regions implicated as being informative regarding response time is consistent with GLM-based fMRI studies [61]. With respect to the subject's decisional variable, the largest information values were observed for the superior frontal and the pre-central gyrus. This implicates a shift of stimulus categorical information towards more frontal regions in comparison to the representation of physical stimulus category discussed above. The pairwise comparison of the superior frontal gyrus with the insular cortex, right intra-parietal, fusiform gyrus and cuneus reached statistical significance (p<0.05) while no overall main effect of region of interest was observed (F_(4.5, 53.7)_ = 1.0, p = 0.38). While it is tempting to speculate that this indicates a more high level cognitive (rather than low level perceptual) determination of the decision on low coherence trials, it should be noted that physical and perceptual stimulus attributes were not completely dissociated in the current study.

In summary, the following picture of spatial representation of information for visual perceptual decisions emerges: with respect to the stimulus, information about differences in stimulus informativeness appears to be represented in a distributed manner throughout the cortical regions studied, while information about the (physical) stimulus category showed a maximum in parietal cortices. Occipito-parietal areas were implicated in the representation of information about the observer's attentional state, while no clear pattern emerged with respect to the speed of the response. On low coherence trials, the superior frontal gyrus was most informative about the observer's decision.

#### Spatiotemporal information representation

Figure 8 displays the spatiotemporal information surfaces related to the external, internal and behavioural state variables for the combined EEG (*R_1_*) and fMRI (*R_2_*) feature variables of interest. The information estimates 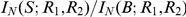 for the respective EEG time-window x fMRI region-of-interest pairings are depicted as the tiles of the surfaces. The regions of interest of each panel are ordered according to an approximate occipital - frontal (or “early sensory - higher cognition”) gradient from left to right, in line with Figure 7 and the time windows of interest according to an early - late gradient from bottom to top.

Each of the information data points (group average, n = 13) has been estimated from the respective joint distribution 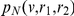, where *v* represents the state variable of interest, *r_1_* the EEG amplitude in the respective time-window and *r_2_* the fMRI signal amplitude for the respective region of interest. It is hence determined by the signal features' joint distribution, i.e. both dependencies between the signal features and the variable of interest and the dependencies between the signal features (both stimulus conditional and non-conditional) themselves.

Overall, the results implicate a complex spatiotemporal pattern of information representation for perceptual decisions in the human brain. For all variables and regions the information estimates are lowest at stimulus onset. Thereafter, information can be observed to flow and accumulate in a distributed manner across time and brain space. The information represented about the stimulus informativeness across all regions of interest differed significantly over time (F_(4,48)_ = 8.8, p<0.001), but not over space (F_(12,144.4)_ = 0.5, p = 0.90). For most regions, the information about the stimulus informativeness over time showed a rebound-pattern: following high early information estimates, a decrease was observed for the 140 ms time-window, followed by a later increase. A significant interaction was not observed (F_(8.4, 101.7)_ = 0.6, p = 0.74). With respect to the regions involved at the final time-point, both high (insula, pre-central) and low (extrastriate visual cortex) areas showed the largest information estimates.

With respect to the perceptual decision task, the most important question concerns the representation of information about the stimulus category on a given experimental trial. The most prominent finding from the spatiotemporal information surface with respect to this variable is the parallel increase of information in both high-level (superior frontal gyrus, frontal eye fields) and low-level (striate, extrastriate cortex) areas over time. This is surprising at least with respect to the low-level areas, as the marginal distributions discussed above did not indicate this. This effect might hence be strongly driven by the joint analysis of occipital electrodes and fMRI regions of interest. Again, the main effect of time-window was significant (F_(2.0, 24.2)_ = 4.2, p = 0.02), the main effect for region of interest and the interaction were not (F_(12, 144)_ = 0.7, p = 0.68, F_(8.3, 99.7)_ = 0.9, p = 0.46, respectively). The largest information estimate for the left frontal eye field was observed at the latest time point considered, i.e., immediately before the initiation of the observer's motor response. Consistent with previous studies implicating the superior frontal gyrus or DLPFC in the representation of a decision variable [25], [26], [47], [62], this region demonstrated a build-up of information over time. Finally, at the latest time-point, the fusiform gyrus was also informative about the stimulus category. However, it did not show the incremental build-up of information seen in the frontal areas. It is tempting to speculate that the observed behaviour of information representation for this low-level area could be explained by recurrent feedback from higher areas [3], [63].

With respect to the internal state, the information surface indicates that early in the decision process mostly low and mid-level cortical areas (extra-striate, the cuneus and the anterior cingulate gyrus) were involved, while later in the decision process both low and high level areas were implicated. Again, the main effect of time-window was significant (F_(4,48)_ = 7.8, p<0.001), but not the main effect of regions of interest or the interaction (F_(5.2,68.9)_ = 0.8, p = 0.50, F_(7.6,90.9)_ = 0.7, p = 0.65, respectively).

Three areas, the extrastriate visual cortex, the cuneus and the left frontal eye field were implicated in the representation of information about the response time throughout the decision process, indicating a sustained process involved for response speed in these areas. Overall, the main effect of time-window was significant (F_(4,48)_ = 7.9, p = <0.001), the main effect of region of interest and the interaction were not (F_(3.1,38.3)_ = 0.43, p = 0.74, F_(7.5,90.5)_ = 0.74, p = 0.64, respectively).

Finally, comparing the representation of the decisional state to the representation of the physical stimulus category showed some differences: firstly, for the physical stimulus category, both high and low level areas showed larger information estimates both early and late during the decision process, while for the observer's decisional variable, this effect was stronger for the high level areas (superior frontal gyrus and left frontal eye field). Interestingly, the most positive deflection for some areas was observed for mid-latency time-windows, as for example in the anterior cingulate and the fusiform gyrus. However, given the supra-threshold nature of the experimental design, a clear dissociation between the subjects' perceptual state and the physical stimulus property at low spatial coherence cannot be obtained in the framework of the current study. Statistical evaluation revealed the usual pattern of significant main effect of time-window (F_(4,48)_ = 4.1, p = 0.005) and non-significant effects of region of interest (F_(4.8, 58.3)_ = 0.5, p = 0.75) and interaction (F_(7.7, 92.7)_ = 1.3, p = 0.24).

In summary, the following picture of spatiotemporal representation of information for visual perceptual decisions emerges: with respect to the external state variables, both low and high level cortical areas were involved in the representation of information with a temporal rebound pattern mainly observed for the informativeness of the stimulus. For the stimulus category, both high and low level areas increased their information content over time, the specific areas being complementary to those implicated in the representation of stimulus informativeness. Regarding the representation of information about the observer's internal state, additional mid-level cortical areas were of relevance. A set of three brain regions was informative about the observer's response time throughout the decision process. Finally, with respect to the categorical decision, the data indicate a stronger involvement of high level cortical areas over time compared to the representation of the physical stimulus category, which implicated both higher/anterior and lower/posterior areas.

## Discussion

This study supports the view that the brain represents information about external, internal and behavioural states in a highly distributed, parallel and dynamic manner. No single brain region or single time-point in the first 500 ms of the perceptual decision process was identified to be of sole relevance. In general, most information was represented in both low (visual cortex) and high (frontal cortex) level regions towards the time of the execution of the decision, with the possible exception of information about the internal state. Finally, some dissociation between the representation of the physical stimulus category and the observer's perceptual interpretation could be identified with a shift of information representation to higher cortical areas in the latter.

What does the current study add with respect to previous studies on visual perceptual decision making? Firstly, in employing an information theoretic framework, the emphasis of the current study is on the information that is represented in the neuronal response on the single-trial level, not averaged over multiple observations. It is hence describing the perceptual decision process at the ecologically most meaningful level, as the brain has to make optimal decisions upon single representation of the perceptual evidence. Secondly, using simultaneous EEG-fMRI recordings, the current study uses state-of-the-art brain imaging methodology to assess the joint EEG-fMRI signal feature probability distributions. Most previous studies, with the exception of [27], employed non-simultaneous or single modality data acquisition schemes and hence are susceptible to between session effects, such as changes in the observer's vigilance, attention, or learning effects. While not a focus of the current communication, future evaluations of the same data set will allow to gain insight into the between-modality dependencies that contribute to information encoding [14]. Here, the degree of the multimodal link directly affects the degree of synergy and redundancy a given feature combination provides with respect to the independent variable of interest. However, at this point, the estimation of synergy is too unreliable to be derived by a simple histogram based probability mass function estimation approach, which is why it is not assessed in the current study. Nevertheless, the principled approach to the joint analysis of EEG-fMRI data opens the door to the investigation of these important questions. Thirdly, the current study explicitly manipulated the observer's internal state by adding a spatial prioritization/attention component to the perceptual decision process. In [26], the authors proposed a spatiotemporal diagram of the processes involved in perceptual decision making based on an EEG-informed analysis of fMRI data. The current study proposes the following additions to this scheme: a) the regions implicated in early temporal visual perception are modulated by the observer's internal state and represent both top-down and bottom-up factors related to perceptual decisions and b) the implication of higher cortical areas in the representation about the observer's decision emphasizes the idea of recurrent feedback loops in the entire network.

Some notes of caution for the interpretation of the results of this study are necessary. First and foremost, the problem of information bias correction for EEG-fMRI experiments remains unresolved. Reasonable precautions not to overestimate information based on the PT-, shuffling-, and null model-correction schemes have been taken in this study. Unfortunately, this highly conservative subtraction procedure occasionally results in theoretically impossible negative information estimates (e.g. for 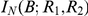). Importantly, however, the focus of the current study is on the relative information content between different signal features and feature combinations, which is robust to shifts in the absolute information baseline [41]. Nevertheless, future applications of the information theoretic framework to EEG-fMRI data sets should strive to optimize existing discrete-data entropy estimation procedures for the specifics of continuous signals [64]. Secondly, any analysis of univariate features is sensitive to the feature selection process. Here, a route informed by the signals' group averages was taken. For some of the comparisons, namely those in which the selection criteria were not orthogonal to the comparison of interest, this entails the danger of circular analysis, which was noted in the discussion of the results [65]. Thirdly the focus of this study was on the amplitude of signal features in the time-domain, and many other features (e.g., EEG frequency components or HRF basis function parameters) are conceivable. Additionally, only the first 500 ms of the perceptual decision process were assessed, and often the highest information estimates were obtained for the final time-window. The focus on the first 500 ms is partly justified by the fact that the observer's response has generally been made by this time-point and by the behaviour of the grand-average ERP, which returns to approximately baseline at this time. However, working memory and error monitoring processes following the decision and response presumably require information representation about the perceptual decision process. Future studies might elucidate the brain's spatiotemporal information representation profile with respect to these. Finally, it can be argued, that other data reduction/feature selection approaches would be more suited to the information theoretic approach for EEG-fMRI. However, for the current study we reasoned that, because the information theoretic approach is not an established framework, for its validation in the context of cognitive paradigms it is first sensibly applied to data features which we expect to be involved in the neural representation of decisional processes. As the literature on the neural underpinnings of perceptual decisions is dominated by GLM analyses of fMRI data and electrode space EEG analyses, these were the primary features we worked with. Only if the IT framework reproduces results comparable to earlier findings can it be sensibly suggested and applied as a stand-alone method for EEG-fMRI analyses. This demonstration is exactly what the current manuscript is provides.

In conclusion, the current study extends our previous experimental validation of the EEG-fMRI information theoretic approach to the cognitive neuroscientific domain and reinforces the notion of brain networks being dynamically involved in the representation of task-relevant information for perceptual decisions. Finally, the information theoretic results provide a guide for the future development of comprehensive forward models for the analysis of simultaneous EEG-fMRI data and a constraint for the spatiotemporal complexity these models will need to achieve.

## Supporting Information

Figure S1 **Eye-movement data.** Eye-movement data were recorded from 8 observer's partaking in the combined EEG-fMRI data acquisition using the long-range ASL 6000 Eye-tracker (Applied Science Laboratories, Bedford, MA) at a sampling frequency of 60 Hz. Eye-tracking data was exported using the Eyenal software (Applied Science Laboratories, Bedford, MA) and imported into Matlab (The Mathworks, Natick, MA). For each subject, samples for which both the pupil circumference and the corneal reflex were not detected were excluded from further analysis. These samples correspond to blinks and recording setup noise. Two observers were excluded from further analysis as the number of invalid samples was too substantial. For the remaining subjects, the session time-series was partitioned into experimental trials comprising the onset of the attention cue (arrow) at 0 seconds, the onset of the stimulus at 1 second and the remaining post-stimulus 2 second period. Mean eye-movement traces around fixation (corrected to 0 degree of visual angle) are shown in Figure S1.A for the stimulus conditions and S1.B for left- and right-hemifield trials, respectively. Data are displayed for both the horizontal and the vertical eye-position (upper panels). Additionally, Figures S1.A and S1.B display the SEM across trials averaged over observers for both horizontal and vertical eye position (lower panels). For none of the eye-position time-series systematic variability upon the onset of the prioritization cue (at 0 s) or stimulus (at 1 s) could be detected, indicating steady fixation throughout the experimental trial. It should be noted that the centre of the peripherally presented stimulus was at 11 degrees of visual angle. Towards the end of the time-series investigated, the variability of the vertical eye position increased slightly, potentially indicating eye-blinks. Based on these data it is unlikely that observer's did not maintain steady fixation and condition specific effects could be explained by eye-movements.
(DOCX)

Figure S2 **Psychophysical pilot study.** To establish that the given stimulus and behavioural manipulations of the perceptual decision task discussed in ‘Materials and Methods’ was successful in evoking a differential behavioural response pattern (response times and accuracy effects), a psychophysical pilot study according to the specification in ‘Materials and Methods’ for the EEG only recordings was conducted with 9 participants (mean age 27.3 years, range 22–37 years). Three of the participants also participated in the main EEG-fMRI experiment approximately four months later. The results of the pilot psychophysical study are shown in Figure S2. As for the main experiment, an increase in stimulus informativeness and spatial prioritization of the stimulus' location led to faster response times and higher response accuracy. Specifically, a two-way repeated measures ANOVA for the median response times including all trials revealed a significant main effect of stimulus coherence (F_(1,8)_ = 20.6, p = 0.002), a significant main effect of prioritization (F_(1,8)_ = 8.3, p = 0.02) and no significant interaction (F_(1,8)_ = 1.9, p = 0.21). Similarly, for median response times on correct response trials only, a significant main effect of stimulus coherence (F_(1,8)_ = 22.2, p = 0.002), a significant main effect of prioritization (F_(1,8)_ = 7.8, p = 0.02) and no significant interaction (F_(1,8)_ = 1.8, p = 0.21) were detected. Finally, for response accuracy, a two-way repeated measures ANOVA revealed a significant main effect of stimulus coherence (F_(1,8)_ = 22.6, p = 0.001), a significant main effect of prioritization (F_(1,8)_ = 3.2, p = 0.11) and no significant interaction (F_(1,8)_ = 2.0, p = 0.19). The paradigm was hence judged adequate for the subsequent EEG-fMRI data acquisition.
(DOCX)

Figure S3 **Feature Extraction.** The information theoretic analyses reported in the current study capitalise on the evaluation of the probability distributions of signal features. These distributions are estimated non-parametrically from the extracted single-trial feature data. Figure S3.A displays an example for a single subject and shows the single-trial time-courses for the electrode and brain regions from which the single-trial estimates were obtained. Inspection of the plots indicates that on most individual trials, a reliable ERP/HRF could be observed. As the last column of averages indicates, the profiles of potential deflections across conditions vary over electrodes, but are qualitatively similar. Likewise, Figure S3.B displays the extracted feature distributions across the experimental conditions. As can be seen, the distributions for the respective features overlap. Similarly, Figures S3.C and S3.D display the extracted feature distributions across the experimental conditions for the fMRI modality in an analogous manner to Figures S3.A and S3.B.
(DOCX)
