# Supplementary material for: EEG-fMRI Based Information Theoretic Characterization of the Human Perceptual Decision System
Source: PLoS One. 2012 Apr 2;7(4):e33896. doi: 10.1371/journal.pone.0033896 (PMC3317669; doi:10.1371/journal.pone.0033896)
Supplement: Figure S1 — Eye-movement data. Eye-movement data were recorded from 8 observer's partaking in the combined EEG-fMRI data acquisition using the long-range ASL 6000 Eye-tracker (Applied Science Laboratories, Bedford, MA) at a sampling frequency of 60 Hz. Eye-tracking data was exported using the Eyenal software (Applied Science Laboratories, Bedford, MA) and imported into Matlab (The Mathworks, Natick, MA). For each subject, samples for which both the pupil circumference and the corneal reflex were not detected were excluded from further analysis. These samples correspond to blinks and recording setup noise. Two observers were excluded from further analysis as the number of invalid samples was too substantial. For the remaining subjects, the session time-series was partitioned into experimental trials comprising the onset of the attention cue (arrow) at 0 seconds, the onset of the stimulus at 1 second and the remaining post-stimulus 2 second period. Mean eye-movement traces around fixation (corrected to 0 degree of visual angle) are shown in Figure S1.A for the stimulus conditions and S1.B for left- and right-hemifield trials, respectively. Data are displayed for both the horizontal and the vertical eye-position (upper panels). Additionally, Figures S1.A and S1.B display the SEM across trials averaged over observers for both horizontal and vertical eye position (lower panels). For none of the eye-position time-series systematic variability upon the onset of the prioritization cue (at 0 s) or stimulus (at 1 s) could be detected, indicating steady fixation throughout the experimental trial. It should be noted that the centre of the peripherally presented stimulus was at 11 degrees of visual angle. Towards the end of the time-series investigated, the variability of the vertical eye position increased slightly, potentially indicating eye-blinks. Based on these data it is unlikely that observer's did not maintain steady fixation and condition specific effects could be explained by eye- [file pone.0033896.s001.docx]

**
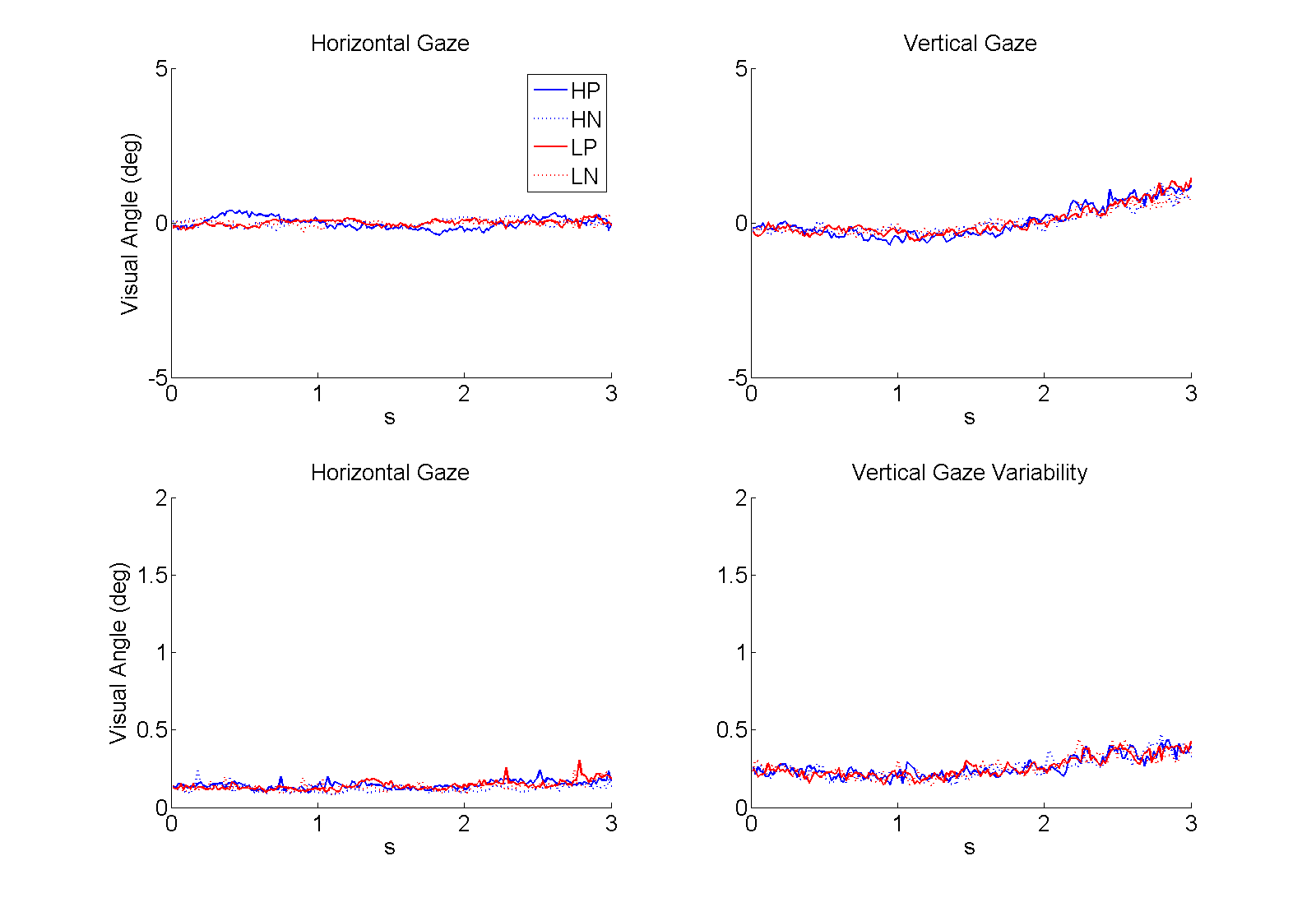
**

Figure S1 A Eye-movements according to stimulus condition. The panels display the eye-tracking time-series of a 3 s peri-stimulus time-window, corresponding to the onset of the prioritization cue at 0 s and the stimulus onset at 1 s. The two upper panel display the mean (n = 6) eye-position in degrees of visual angle for the horizontal and vertical eye-position. The lower two panels display the mean (n = 6) SEM of the eye-position across trials (HP: High informativeness, prioritized, HN: High Informativeness, not prioritized, LP: Low informativeness, prioritized, LN: Low informativeness, not prioritized)

**
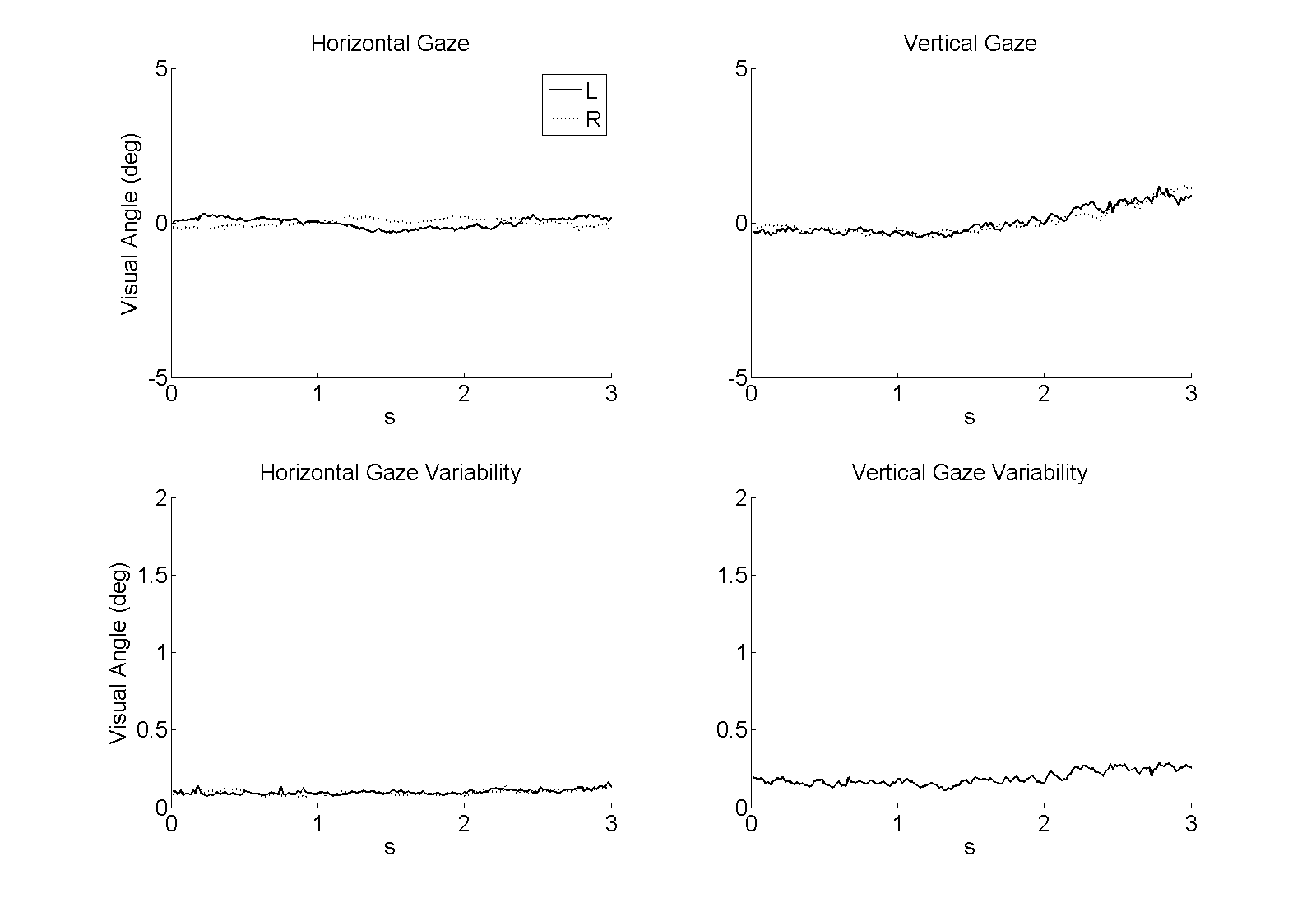
**

Figure S1 B Eye-movements according to stimulus presentation side. The panels display the eye-tracking time-series of a 3 s peri-stimulus time-window, corresponding to the onset of the prioritization cue at 0 s and the stimulus onset at 1 s. The two upper panel display the mean (n = 6) eye-position in degrees of visual angle for the horizontal and vertical eye-position. The lower two panels display the mean (n = 6) SEM of the eye-position across trials (L: left hemifield trials, R: right hemifield trials)
