# Supplementary material for: EEG-fMRI Based Information Theoretic Characterization of the Human Perceptual Decision System
Source: PLoS One. 2012 Apr 2;7(4):e33896. doi: 10.1371/journal.pone.0033896 (PMC3317669; doi:10.1371/journal.pone.0033896)
Supplement: Figure S2 — Psychophysical pilot study. To establish that the given stimulus and behavioural manipulations of the perceptual decision task discussed in ‘Materials and Methods’ was successful in evoking a differential behavioural response pattern (response times and accuracy effects), a psychophysical pilot study according to the specification in ‘Materials and Methods’ for the EEG only recordings was conducted with 9 participants (mean age 27.3 years, range 22–37 years). Three of the participants also participated in the main EEG-fMRI experiment approximately four months later. The results of the pilot psychophysical study are shown in Figure S2. As for the main experiment, an increase in stimulus informativeness and spatial prioritization of the stimulus' location led to faster response times and higher response accuracy. Specifically, a two-way repeated measures ANOVA for the median response times including all trials revealed a significant main effect of stimulus coherence (F(1,8) = 20.6, p = 0.002), a significant main effect of prioritization (F(1,8) = 8.3, p = 0.02) and no significant interaction (F(1,8) = 1.9, p = 0.21). Similarly, for median response times on correct response trials only, a significant main effect of stimulus coherence (F(1,8) = 22.2, p = 0.002), a significant main effect of prioritization (F(1,8) = 7.8, p = 0.02) and no significant interaction (F(1,8) = 1.8, p = 0.21) were detected. Finally, for response accuracy, a two-way repeated measures ANOVA revealed a significant main effect of stimulus coherence (F(1,8) = 22.6, p = 0.001), a significant main effect of prioritization (F(1,8) = 3.2, p = 0.11) and no significant interaction (F(1,8) = 2.0, p = 0.19). The paradigm was hence judged adequate for the subsequent EEG-fMRI data acquisition. (DOCX) [file pone.0033896.s002.docx]

**
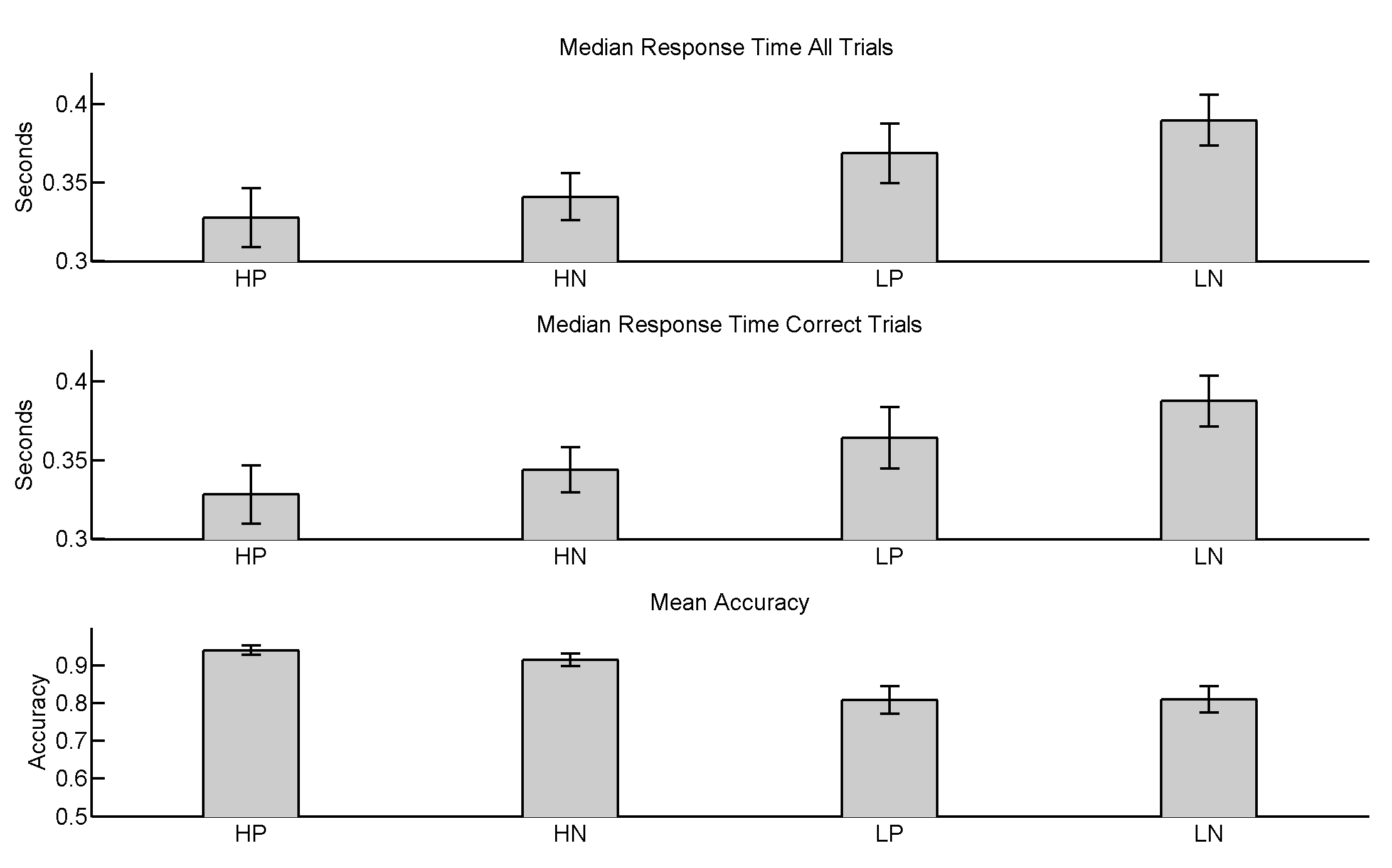
**

Figure S2 Psychophysical pilot study results. Uppermost panel: Average median response time for all experimental trials across observers ± SEM. Middle panel: Average median response time for correct response trial across observers ± SEM. Lowermost panel: Average response accuracy across observers ± SEM (HP: High Informativeness, Prioritized, HN: High Informativeness, Not Prioritized, LP: Low Informativeness, Prioritized, LN: Low Informativeness, Not Prioritized
