# Supplementary material for: EEG-fMRI Based Information Theoretic Characterization of the Human Perceptual Decision System
Source: PLoS One. 2012 Apr 2;7(4):e33896. doi: 10.1371/journal.pone.0033896 (PMC3317669; doi:10.1371/journal.pone.0033896)
Supplement: Figure S3 — Feature Extraction. The information theoretic analyses reported in the current study capitalise on the evaluation of the probability distributions of signal features. These distributions are estimated non-parametrically from the extracted single-trial feature data. Figure S3.A displays an example for a single subject and shows the single-trial time-courses for the electrode and brain regions from which the single-trial estimates were obtained. Inspection of the plots indicates that on most individual trials, a reliable ERP/HRF could be observed. As the last column of averages indicates, the profiles of potential deflections across conditions vary over electrodes, but are qualitatively similar. Likewise, Figure S3.B displays the extracted feature distributions across the experimental conditions. As can be seen, the distributions for the respective features overlap. Similarly, Figures S3.C and S3.D display the extracted feature distributions across the experimental conditions for the fMRI modality in an analogous manner to Figures S3.A and S3.B. (DOCX) [file pone.0033896.s003.docx]

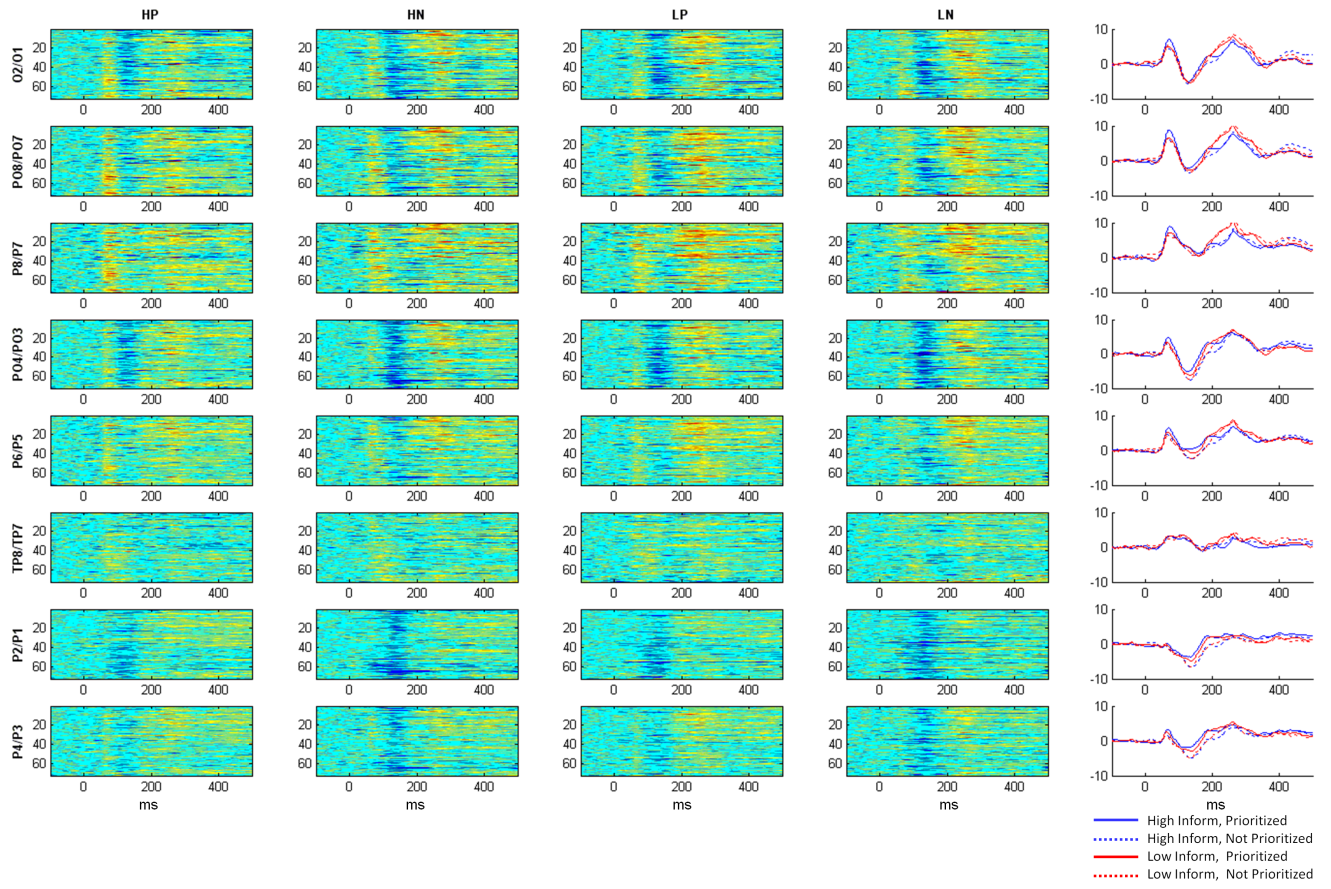


Figure S3 A Single subject single-trial EEG plots. The rightmost column displays the experimental condition averages with the same colour coding as in Figure 6.6. (HP: High Informativeness, Prioritized, HN: High Informativeness, Not Prioritized, LP: Low Informativeness, Prioritized, LN: Low Informativeness, Not Prioritized).


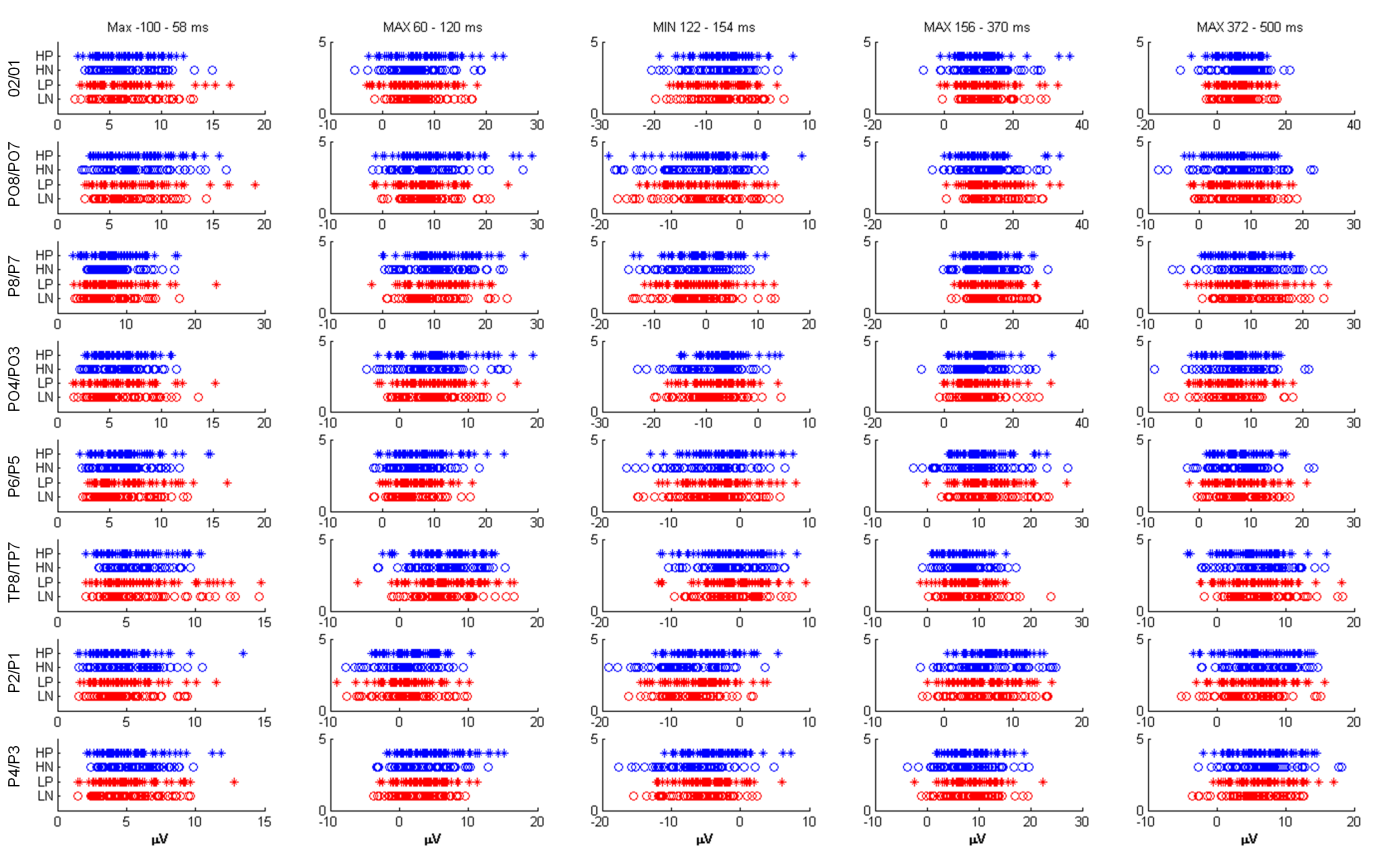


Figure S3 B Single subject EEG feature distributions. The y-axis annotation represent the four experimental conditions (HP: High Informativeness, Prioritized, HN: High Informativeness, Not Prioritized, LP: Low Informativeness, Prioritized, LN: Low Informativeness, Not Prioritized).


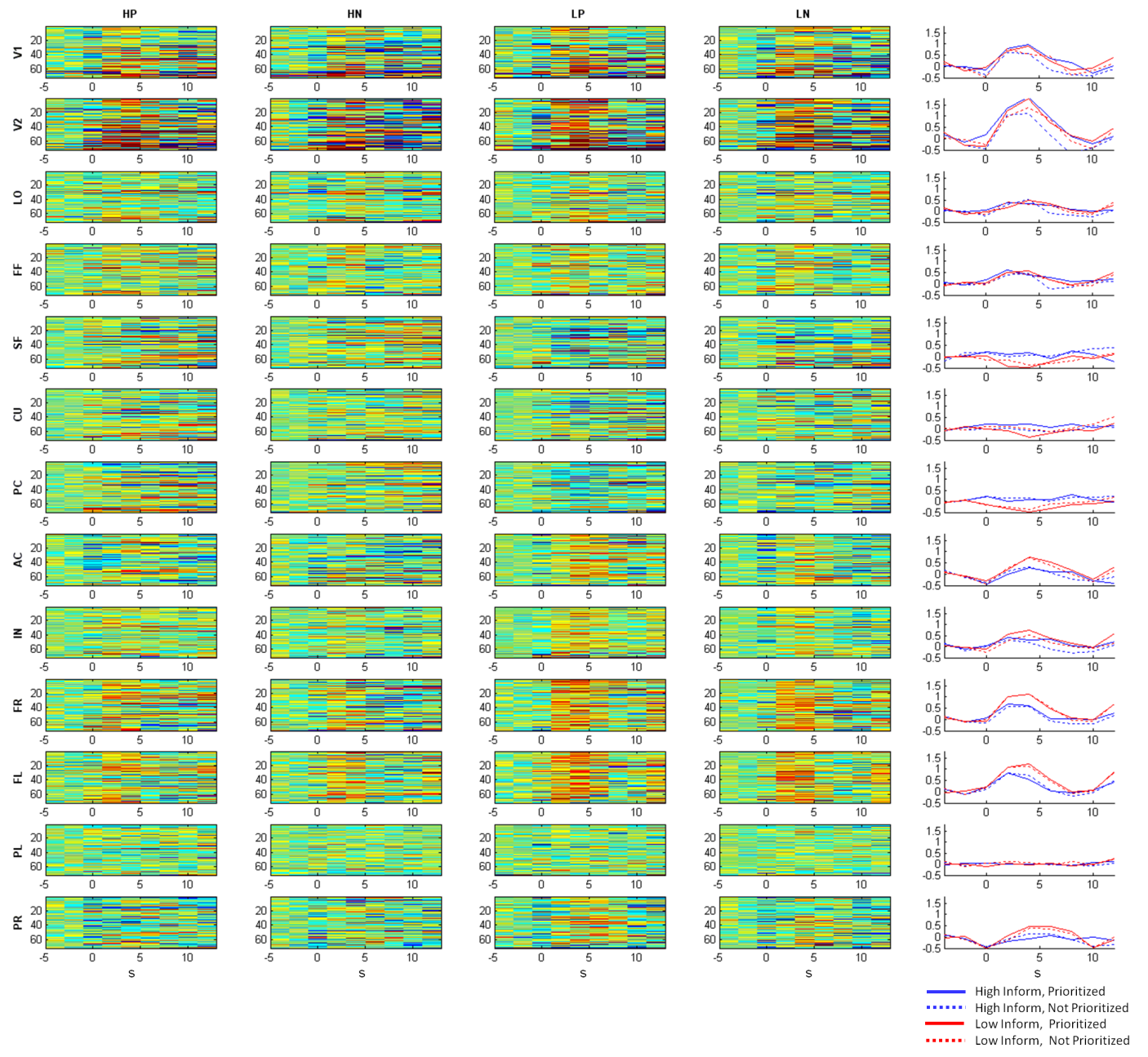


Figure S3 C Single subject single-trial fMRI plots. The rightmost column displays the experimental condition averages with the same colour coding as in Figures 6.8 and 6.9. (HP: High Informativeness, Prioritized, HN: High Informativeness, Not Prioritized, LP: Low Informativeness, Prioritized, LN: Low Informativeness, Not Prioritized. The ROIs are abbreviated as V1: Striate Cortex, V2: Extrastriate Cortex, LO: Lateral Occipital Complex, CU: Cuneus, FF: Fusiform Gyrus, PL: L. Intra-Parietal Sulcus, PR: R.Intra-Parietal Sulcus, PC:Post-Central Gyrus, AC: R.Anterior Cingulate, IN:R. Insula, FL:L. Frontal Eye Field, FR: R. Frontal Eye Field, SF: L. Superior Frontal Gyrus)


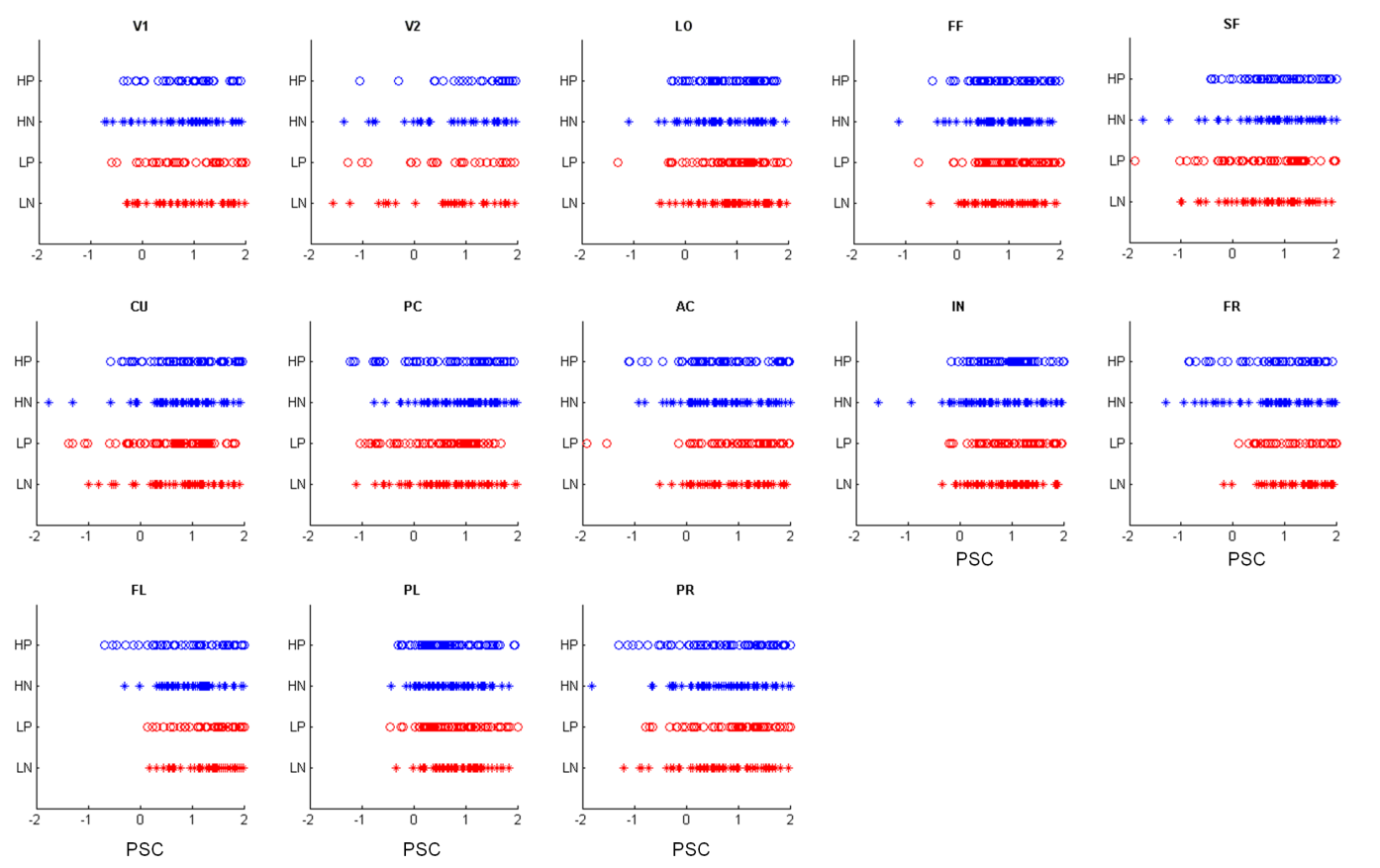


Figure S3 D Single subject fMRI feature distributions. The y-axis annotation represent the four experimental conditions (HP: High Informativeness, Prioritized, HN: High Informativeness, Not Prioritized, LP: Low Informativeness, Prioritized, LN: Low Informativeness, Not Prioritized. The ROIs are abbreviated as V1: Striate Cortex, V2: Extrastriate Cortex, LO: Lateral Occipital Complex, CU: Cuneus, FF: Fusiform Gyrus, PL: L. Intra-Parietal Sulcus, PR: R.Intra-Parietal Sulcus, PC:Post-Central Gyrus, AC: R.Anterior Cingulate, IN:R. Insula, FL:L. Frontal Eye Field, FR: R. Frontal Eye Field, SF: L. Superior Frontal Gyrus, PSC: percent signal change)
